# Development of novel antimicrobials with engineered endolysin LysECD7-SMAP to combat Gram-negative bacterial infections

**DOI:** 10.1186/s12929-024-01065-y

**Published:** 2024-07-24

**Authors:** Daria V. Vasina, Nataliia P. Antonova, Vladimir A. Gushchin, Andrey V. Aleshkin, Mikhail V. Fursov, Anastasiia D. Fursova, Petya G. Gancheva, Igor V. Grigoriev, Pavel Grinkevich, Alexey V. Kondratev, Alexey V. Kostarnoy, Anastasiya M. Lendel, Valentine V. Makarov, Maria A. Nikiforova, Andrei A. Pochtovyi, Tatiana Prudnikova, Timofey A. Remizov, Natalia V. Shevlyagina, Andrei E. Siniavin, Nina S. Smirnova, Alexander A. Terechov, Artem P. Tkachuk, Evgeny V. Usachev, Aleksei M. Vorobev, Victoria S. Yakimakha, Sergey M. Yudin, Anastasia A. Zackharova, Vladimir G. Zhukhovitsky, Denis Y. Logunov, Alexander L. Gintsburg

**Affiliations:** 1 N.F. Gamaleya National Research Centre for Epidemiology and Microbiology, Ministry of Health of the Russian Federation, Moscow, Russia; 2 Faculty of Biology, Lomonosov Moscow State University, Moscow, Russia; 3 G.N. Gabrichevsky Moscow Research Institute for Epidemiology and Microbiology, Moscow, Russia; 4 State Research Center for Applied Microbiology and Biotechnology, Obolensk, Russia; 5 Faculty of Science, University of South Bohemia in Ceske Budejovice, Ceske Budejovice, Czech Republic; 6 Centre for Strategic Planning and Management of Biomedical Health Risks of the Federal Medical Biological Agency, Moscow, Russia; 7 Russian Medical Academy of Continuing Professional Education (RMANPO), Ministry of Public Health, Moscow, Russia; 8 Department of Infectiology and Virology, Federal State Autonomous Educational Institution of Higher Education I. M. Sechenov First Moscow State Medical University of the Ministry of Health of the Russian Federation (Sechenov University), Moscow, Russia

**Keywords:** Engineered endolysin, Gram-negative bacteria, Preclinical efficacy, Dosage forms, Enzyme-based antibacterial

## Abstract

**Background:**

Among the non-traditional antibacterial agents in development, only a few targets critical Gram-negative bacteria such as carbapenem-resistant *Pseudomonas aeruginosa*, *Acinetobacter baumannii* or cephalosporin-resistant *Enterobacteriaceae*. Endolysins and their genetically modified versions meet the World Health Organization criteria for innovation, have a novel mode of antibacterial action, no known bacterial cross-resistance, and are being intensively studied for application against Gram-negative pathogens.

**Methods:**

The study presents a multidisciplinary approach, including genetic engineering of LysECD7-SMAP and production of recombinant endolysin, its analysis by crystal structure solution following molecular dynamics simulations and evaluation of antibacterial properties. Two types of antimicrobial dosage forms were formulated, resulting in lyophilized powder for injection and hydroxyethylcellulose gel for topical administration. Their efficacy was estimated in the treatment of sepsis, and pneumonia models in BALB/c mice, diabetes-associated wound infection in the leptin receptor-deficient db/db mice and infected burn wounds in rats.

**Results:**

In this work, we investigate the application strategies of the engineered endolysin LysECD7-SMAP and its dosage forms evaluated in preclinical studies. The catalytic domain of the enzyme shares the conserved structure of endopeptidases containing a putative antimicrobial peptide at the C-terminus of polypeptide chain. The activity of endolysins has been demonstrated against a range of pathogens, such as *Klebsiella pneumoniae, A. baumannii, P. aeruginosa*, *Staphylococcus haemolyticus, Achromobacter spp, Burkholderia cepacia complex* and* Haemophylus influenzae,* including those with multidrug resistance. The efficacy of candidate dosage forms has been confirmed in in vivo studies. Some aspects of the interaction of LysECD7-SMAP with cell wall molecular targets are also discussed.

**Conclusions:**

Our studies demonstrate the potential of LysECD7-SMAP therapeutics for the systemic or topical treatment of infectious diseases caused by susceptible Gram-negative bacterial species and are critical to proceed LysECD7-SMAP-based antimicrobials trials to advanced stages.

**Supplementary Information:**

The online version contains supplementary material available at 10.1186/s12929-024-01065-y.

## Background

The high emergence of bacterial infections caused by antimicrobial-resistant pathogens, particularly Gram-negative pathogen-associated infections, poses an increasing threat to human health. ESKAPE pathogens, such as *Enterobacteriaceae*, *Acinetobacter baumannii*, *Pseudomonas aeruginosa*, are capable of acquiring multiple resistance to antibiotics, including the most effective carbapenems and third-generation cephalosporins, and cause the most severe consequences of infection, particularly in healthcare settings. Several innovative therapeutic concepts have been proposed to address this global problem, including phages and phage-derived proteins [1, 2].

Endolysins are bacteriophage lytic enzymes that can hydrolyze cell wall peptidoglycan, causing lysis and bacterial cell death. Recombinant endolysins and their genetically engineered derivatives are attractive antibacterials because of their proven in vitro and potential in vivo efficacy in the treatment of various bacteremia and local bacterial infections [3–5]. These enzymes are specific, fast-acting, effective against antimicrobial-resistant bacteria, capable of breaking down bacterial biofilms, and also environmentally friendly because, unlike antibiotics, they do not contribute to environmental pollution. [3, 6]. Despite the claimed therapeutic benefits of endolysins, there is limited evidence of their efficacy and safety in preclinical animal studies and clinical trials in healthy volunteers and patients [7]. Existing data, particularly for enzymes targeting Gram-negative bacteria (*Pseudomonas*, *Klebsiella*, *Acinetobacter*, etc.), are limited to efficacy studies in animal models [8–11], while preclinical studies have yet to be translated into relevant clinical trials to demonstrate the potential of this new class of antibacterial drugs.

For intravenous endolysin-based drugs, there are almost no specific formulation strategies available in the literature [5], with the exception of nanocarriers (nanostructure or nanocomposite assembly). Many of the approaches focus on protein engineering, to prolong the endolysin half-life and improve PK/PD. For topical application, several formulation strategies have been considered, most of which have been presented by different gel forms containing additional anti-inflammatory and wound healing compounds [5]. Water soluble gels are a promising approach as they maintain enzyme activity and provide the wound hydration necessary for the wound healing process. However, most animal studies are performed with endolysins protein solutions without any formulation [12–15], and we still lack information on the characteristics of the dosage forms and the effect of the composition on the efficacy, safety and stability of drug candidates.

Previously, we have described LysECD7 endolysin modified with an optimized fragment of the permeabilizing peptide SMAP-29 [16]. This fusion showed improved in vitro activity against Gram-negative bacteria and stability characteristics compared to the parent enzyme. Based on these studies, we consider this hybrid protein to be promising for further antimicrobials development. Here we describe the results of extensive in vitro and in vivo studies of LysECD7-SMAP, including the development and evaluation of the therapeutic efficacy of dosage forms for parenteral and topical administration.

## Methods

### Bacterial strains

The bacterial strains used in the study included laboratory strains and clinical isolates of Gram-negative and Gram-positive pathogens from the collection of the Gamaleya National Center for Epidemiology and Microbiology (Russia), the State collection of pathogenic microorganisms and cell cultures “SCPM-O-B” and the collection of Gabrichevsky Moscow Research Institute of Epidemiology and Microbiology.

For animal studies *K. pneumoniae* M9 and *P. aeruginosa* 38–16 isolates were used. Strains were cultivated in GRM-1 media (pancreatic hydrolysates of fish meal (15.0 g/L) and casein (10.0 g/L), yeast extract (2.0 g/L), NaCl (3.5 g/L), glucose (1.0 g/L), SRCAMB) at 37 °C, at 240 rpm overnight. Antibiotic susceptibility testing of these strains was performed by the broth microdilution method using Mueller–Hinton broth (Thermo Fisher Scientific), according to ISO recommendations [17]. Results were interpreted according to The European Committee on Antimicrobial Susceptibility Testing. Breakpoint tables for interpretation of MICs and zone diameters. Version 12.0, 2022 (https://www.eucast.org).

### Protein expression and purification

LysECD7 was obtained as previously described [16]. To express LysECD7-SMAP its coding sequence (Additional file 1) was artificially synthesized in a pAL-TA commercial vector (Evrogen Ltd.). Thereafter endolysin ORF was amplified from a pALTA-LysECD7-SMAP clone and integrated into the expression vector pET-42b (+) (Evrogen Ltd.), resulting in a pET42b-LysECD7-SMAP plasmid (kanamycin resistance). All constructs were checked for errors via Sanger sequencing.

The expression vector was introduced into the competent *Escherichia coli* strain BL21 (DE3) pLysS (chloramphenicol resistance) using a heat shock transformation protocol. The *E. coli* were grown in LB broth (37 °C, 240 rpm) to OD_600_ = 0.55–0.65 and then induced with 1 mM β-D-1-thiogalactopyranoside (IPTG) at 37 °C for 4 h. The cells were harvested by centrifugation (6000 × *g* for 20 min at 4 °C) and resuspended in a lysis buffer (20 mM Tris HCl, 250 mM NaCl, 0.1 mM EDTA, 1 mM PMSF pH 8.0). Then, the suspension was disrupted by APV Laborhomogenisierer 2000 (SPX FLOW, Inc.) and the cell debris was removed by centrifugation (10,000 × *g* for 30 min at 4 °C). The supernatant was filtered through a 0.2 μm filter and diluted fivefold with 0.36 M NaCl, 50 mM Tris–HCl pH 7.5.

The protein was purified using ÄKTA avant 150 system with XK 50/30 Column (GE Healthcare), prepacked with Capto SP ImpRes resin (GE Healthcare). The cell lysate was applied to a column pre-equilibrated with binding buffer (0.36 M NaCl, 50 mM Tris–HCl pH 7.5). Then, the column was washed with the binding buffer. The protein fractions were eluted using a linear gradient to a 100% elution buffer (0.55 M NaCl, 50 mM Tris–HCl pH 7.5). The collected protein fractions were concentrated with Labscale TFF System (Merck-Millipore) supplied with Pellicon^®^ XL50 Ultracel^®^ 10 kDa membrane (Merck-Millipore).

During the next step, protein solution was purified with XK 50/30 Column, prepacked with Superdex 75 resin (GE Healthcare) and preequilibrated with 0.55 M NaCl, phosphate-buffered saline (PBS) pH 7.4, and, finally, desalted on HiPrep^™^ 26/10 Desalting column (GE Healthcare) to PBS pH 7.4 buffer. LysECD7-SMAP fractions were collected and concentrated with Pellicon^®^ XL50 Ultracel^®^ 10 kDa membrane to protein concentration 20 mg ml^−1^ and sterilized by 0.2 μm filtration.

The purity of the protein was determined by 16% SDS-PAGE (molecular mass 17.2 kDa). The protein concentration was measured using a spectrophotometer (Implen NanoPhotometer, IMPLEN) at 280 nm and calculated using a predicted extinction coefficient (1.44 (mg/ml)^−1^ cm^−1^).

### Crystallization

Crystallization screening was performed on an Oryx4 crystallization robot (Douglas Instruments Ltd) in MRC 2-well crystallization plates (Hampton Research (HR)) and Combi Clover Junior crystallization plates (Rigaku Reagents), respectively, employing the sitting drop vapour diffusion method. Proteins solutions of LysECD7 in 100 mM Tris–HCl buffer pH 7.5 and LysECD7-SMAP in 100 mM MES buffer pH 9.5 were used for the initial crystallization trials. Commercial crystallization screens were used: JCSG-plus, Structure Screen 1&2, Morpheus, the PGA screen, MIDAS (Molecular Dimensions Ltd) and Index (HR). The proteins with concentrations of 5 mg ml^−1^ and 10 mg ml^−1^ were mixed with precipitant solutions in ratios 1:1 and 2:1, equilibrated against 50–70 µl precipitant solution in the reservoirs at 293 K. Optimization of successful crystallization conditions was performed by variation of salt, PEG and protein concentration [18]. Later on, various additives were used as well as the microseeding technique, aimed to adjust crystal size and quality. 3D crystals of LysECD7 with a period of three weeks were grown under condition containing 0.2 M Ammonium sulfate, 20% w/v Polyethylene glycol 3350 pH 6.0 with concentrations of 5 mg ml^−1^. The LysECD7-SMAP crystals were obtained with the precipitant composed of 0.2 M Potassium sodium tartrate tetrahydrate, 2.0 M Ammonium sulfate and 0.1 M Sodium citrate 5.6 during one month.

### Data collection and processing

X-ray diffraction data for LysECD7 and LysECD7-SMAP at 100 K were collected at the BESSY II electron-storage ring on the MX14.1 beamline operated by the Helmholtz-Zentrum Berlin (Berlin-Adlershof, Germany) to the 2.5 and 1.7 Å resolution, respectively. Data were recorded on the Pilatus detector S3 2 M (Dectris). The dataset was collected on the crystals from different crystallization conditions. 1800 diffraction images each were collected with 0.1 oscillation angle. Collected data were processed using XDS program [19] with the XDSAPP graphical user interface [20].

The search model for LysECD7 protein was generated by ARCIMBOLDO light [21, 22], based on the combination of locating small model fragments with density modification with the program SHELXE [23]. LysECD7 and LysECD7-SMAP crystal structures were determined by automated model-building pipeline using models from BUCCANEER [24]. LysECD7 solved structure was used as a template for the molecular replacement phasing using MOLREP [25]. The structures of LysECD7 and LysECD7-SMAP were further refined by REFMAC5 from the CCP4 package [26] and manually modelled using COOT program package [27]. MolProbity server was used for final model geometry validation. For determination of the protein assembly, PDBePISA [28] was applied. The structures of both proteins were deposited to the Protein Data Bank. For LysECD7 accession code is 8Q2G (LysECD7-SMAP structure is undergoing validation in PDB). Figures with the 3D structure were prepared using the program PyMOL. The complete information about the data collection and refinement statistics are provided in Additional file 1.

For intrinsic disordered regions prediction PONDR^®^ software with VL-XT, VSL2 and VL3-BA algorithms was used (http://www.pondr.com), the calculation and presentation of various sequence parameters associated with disordered protein sequences were provided with CIDER (Classification of Intrinsically Disordered Ensemble Regions (http://pappulab.wustl.edu/CIDER/analysis/). For AMP prediction AxPEP Server with AmPEP and Deep-AmPEP30 instruments (https://cbbio.online/AxPEP/?), AMPfun web server (http://fdblab.csie.ncu.edu.tw/AMPfun/index.html) and Antimicrobial Peptide Scanner vr.2 (https://www.dveltri.com/ascan/v2/ascan.html) were applied.

### Molecular dynamics simulations

PyMOL was used for initial structures editing including amino acids substitution (virtual mutagenesis) and concatenation of structures as well as results visualization. As no SMAP-peptide could be resolved using the obtained protein crystal structure, and LysECD7-SMAP was artificially fused to homologous SMAP structure from PDB ID 5Z2O (RALRRLARKIAHAVKKYG) to produce LysECD7-SMAP model. The modified SMAP used in this study differs in several positions including 3 substitutions and absence of trailing glycine (RKLRRLKRKIAHKVKKY-) thus, the 5Z2O structure was additionally edited to match the target sequence and modified SMAP structure was linked with resulting in initial LysECD7-SMAP model.

Amber16 was used for molecular dynamics simulations and results analysis. Molecular dynamics simulations were performed with Amber ff14SB [29] force field in 3 stages: (1) Energy minimization, 2500 Steepest Descend algorithm steps, 2500 Conjugate Gradient algorithm steps. (2) Heating: 0.002 ps time step, 9000 steps from 0 to 310 K, then 1000 steps at 310 K, Langevin thermostat, no pressure control. (3) Production run: 200 ns total, 2 × 100 ns (50000000 steps) sequental runs, 0.002 ps time step, 310 K, Langevin thermostat, Berendsen barostat, 1 bar pressure control, 8 Å cut-off for non-bonded interactions. The SHAKE algorithm was applied to consider constrain bonds involving hydrogen atoms.

### Antibacterial in vitro activity test

Overnight bacterial cultures of *A. baumannii* (n = 6)*, P. aeruginosa* (n = 12)*, K. pneumoniae* (n = 9)*, B. cepacia complex* (n = 5)*, Achromobacter* spp (n = 4)*, H. influenzae* (n = 4)*, Staphylococcus aureus* (n = 8), and *S. haemolyticus* (n = 3) strains were diluted 30-fold in Mueller–Hinton broth and grown to the exponential phase (OD_600_ = 0.6). Subsequently, the cells were harvested by centrifugation (6000 × *g*, 10 min) and resuspended in the same volume of PBS pH 7.4. Each suspension was diluted 100-fold in the 20 mM Tris–HCl pH 7.5 to a final density of approximately 10^6^ cells/ml. Afterwards, 100 μl of the bacterial suspensions and 100 μl of the protein solutions with 200 µg ml^−1^ concentration (final protein concentration is 100 µg ml^−1^) were mixed in 96-well plates, with a buffer lack of endolysin used as the negative control. The mixtures were incubated at 37 °C for 5, 10, 30, 60 or 120 min to study the kinetics of antibacterial action and for 30 min to study spectrum of activity, with shaking at 200 rpm and then were diluted tenfold in PBS pH 7.4.

Subsequently, 100 μl of each dilution was plated onto Mueller–Hinton agar, and the number of bacterial colonies was counted after an overnight incubation at 37 °C. All experiments were performed in triplicate, and the antibacterial activity was expressed as follows: Antibacterial activity (%) = 100%—(CFUexp/CFUcont) × 100%, where CFUexp is the number of bacterial colonies in the experimental culture plates, and CFUcont is the number of bacterial colonies in the control culture plates. Antibacterial activity was arbitrarily regarded as meaningful when it was higher than 33%. For all strains, three independent replicates were done.

### Antibiofilm activity

*A. baumannii* 50–16 and *K. pneumoniae* biofilm-forming strains were cultured overnight in TSB medium (BD Tryptic Soy Broth). Then culture was diluted 1:50 in fresh TSB, added 100 µl to wells of 96-well sterile polystyrene cell culture plates and incubated for 24 h at 37 °C, 250 rpm to allow biofilm formation. After incubation, the wells’ contents with planktonic cells were shaken off, the plate was washed with 200 µl of PBS pH 7.4 three times and air-dried for approximately 10 min. Then 100 µl of endolysin solutions in final concentration of 100, 1000 µg ml^−1^ or 20 mM Tris–HCl pH 7.5 as a negative control were added to the wells and incubated at 37 °C, 250 rpm for 2 h. After incubation, wells were twice washed with 200 µl of PBS pH 7.4 and air-dried. Washed biofilms were stained with 0.1% aqueous solution of crystal violet (Applichem) for 20 min at RT followed by triple rinsing with PBS. The remaining stain was re-solubilized in 200 µl of 33% acetic acid and OD_590_ of obtained solution was measured using SPECTROstar NANO Absorbance Reader (BMG LABTECH). Alcian blue staining was performed with 1% solution in 3% acetic acid for 30 min and measurement of OD_615_. All experiments were performed in six replicates.

The biofilms formation interpretation was done accordingly to [30]. Briefly, weak biofilm was defined at OD_c_ < OD_Ab_ ≤ 2 × OD_c_, moderate biofilm at 2 × OD_c_ < OD_Ab_ ≤ 4 × OD_c_, and strong biofilm at 4 × OD_c_ < OD_Ab_, where OD_c_ is the cut-off value calculated as three standard deviations (SD) above the mean OD of the negative control, OD_Ab_ = the optical density of *A. baumannii* 50–16 well stained with crystal violet.

### Microscopy

To provide planktonic cells microscopy an overnight *A. baumannii* 50–16 and *S. aureus* 73–14 bacterial cultures were diluted in an LB broth, grown to an exponential phase (OD_600_ = 0.6) and harvested by centrifugation. Subsequently, cell pellets were washed twice with distilled water and resuspended in water to a final density of approximately 10^8^ cells/ml (McFarland 0.5). Afterwards, 150 µl of the bacterial suspensions were mixed with 150 µl of the protein water solutions in to the 10 µg ml^−1^ final concentration and incubated at 37 °C for 30 min with shaking at 200 rpm. Distilled water was used as the negative control. Mixtures were fixed by adding 600 µl of 10% formaldehyde solution for 24 h. Next, 10 µl of mixtures were placed on a slide and dried at room temperature conditions. The samples were mounted to stubs and sputter-coated with a gold layer (5 nm) in an SPI-Module Sputter/Carbon Coater System (SPI Inc.). The sputtering samples were analyzed by means of a scanning dualbeam electron microscope Quanta 200 3D (FEI Company) using the high vacuum mode (10 kV).

### Bacterial biofilms microscopy

Sterile glass circle cover slides (Hampton Research) were plunged into overnight *A. baumannii* 50–16 culture in TSB medium and incubated at 37 °C for 24 h without shaking for biofilm formation. Then slides were washed with distilled water three times and air-dried. Two slides were submerged into 20 mM Tris–HCl pH 7.5 control buffer, another two into 100 µg ml^−1^ LysECD7-SMAP solution for 2 h at room temperature. Afterwards, all slides were again washed with distilled water three times and air-dried. One slide from each group was fixed in 10% formaldehyde vapors for 24 h and analyzed using scanning electron microscopy after coating with a gold layer as described above.

Another two slides were stained with 0.1% aqueous solution of crystal violet for 20 min at room temperature, washed three times with water and dried at room temperature. These slides were analyzed using an Axiostar plus Transmitted Light Microscope (Zeiss AG) at 400 × magnification.

### Peptidoglycan binding assay

Bacterial PG was isolated from *E. coli* M15 strain as described by [31] with modifications. Briefly, *E. coli* cells were grown in LB to OD_600_ = 0.7, centrifuged at 6000 × *g* at 4 °C for 10 min and resuspended in 20 ml of ice-cold water per liter of bacterial culture. Cell’s suspension was poured into an equal volume of 8% SDS solution and stirred in a boiling water bath for 1 h. Sample was cooled to RT and PG was pelleted by ultracentrifugation at 130,000 × *g* at room temperature for 1 h. Resuspended in 20 ml water PG was poured into boiling 8% SDS solution and incubated for 15 min. Then peptidoglycan was washed four times with room temperature water, centrifuged 1 h at 130,000 × *g* for each wash to remove residual SDS. PG was resuspended in 10 ml water, supplied with equal volume of 10 mM Tris–HCl pH 7.0 and 0.12 mg ml^−1^ trypsin and incubated for 45 min to hydrolyze proteins trapped in PG sacculi. This mixture was added to an equal volume of boiling 8% SDS solution and incubated for 15 min in a boiling-water bath. PG was washed four times with water at room temperature as described earlier. Purified PG was resuspended in 1 ml water and stored at 4 °C. PG concentration was determined by OD_206_ measurement according to the standard curve made with *Micrococcus lysodeikticus* PG (Sigma-Aldrich).

The binding of endolysin to PG was assessed after the 30 min incubation of 5 μg protein with peptidoglycan in 10:1, 1:1, 1:10 and 1:20 LysECD7-SMAP:PG ratios at RT for. Mixtures were separated by centrifugation (16,000 × *g*, 15 min, 4 °C) and unbound fractions (supernatant) were analyzed with 16% SDS-PAGE gels following Coomassie brilliant blue (CBB) staining.

Antibacterial activity was assessed after incubation of endolysin (10 μg) and peptidoglycan in 10:1, 1:1, 1:10 and 1:100 LysECD7-SMAP:PG ratios mixtures for 30 min as described above using in vitro activity test with *A. baumannii* 50–16 strain.

### Lipopolysaccharides binding assays

Purified bacterial *Salmonella typhimurium* LPS (#437650, Calbiochem^®^, Merck KGaA) was used in the experiment. LysECD7-SMAP (5 μg) was preincubated with LPS in 1:1 LysECD7-SMAP:LPS ratio at RT for 3 h. Mixtures were separated by centrifugation (16,000 × *g*, 15 min, 4 °C) and supernatants were analyzed on 16% SDS-PAGE gels with CBB staining. LPS-LysECD7-SMAP ELISA binding analysis was done using LPS from *S. typhimurium*, murine monoclonal antibodies IgG1 towards LPS from *S. typhimurium* (#1E6cc, HyTest Ltd.) and HRP-conjugated polyclonal goat-anti mouse IgG (#L20/01, HyTest Ltd.). For this, the wells of polystyrene 96-well plate (Xema-Medica) were coated with 1 µg of LysECD7-SMAP per well in PBS buffer pH 7.4 (VWR Chemicals) at 4 °C for 24 h and blocked with 200 µl of 0.025% BSA for 1 h at 37 °C, 600 rpm. Then, 0.3 µg of *S. typhimurium* LPS was added to each well and inoculated for 1 h at 37 °C, 600 rpm. Incubation was performed in PBS buffers containing 0.05% Triton X100, 1 mM EDTA, and supplemented with 0, 100 or 500 mM NaCl, these buffers were also used as negative controls. After incubation the wells were thrice washed with PBS supplemented with 0.05% Triton X100, and 0, 100 or 500 mM NaCl. Then, murine IgG1 monoclonal antibodies were diluted 1:4000, and 100 µl were added per well and incubated for 1 h at 37 °C, 600 rpm. Wells were washed three times with PBS with 0.05% Triton × 100 and incubated with 100 µl of 1:50000 diluted HRP-conjugated goat anti-mouse IgG polyclonal antibodies (#L20/01, HyTest Ltd.) for 1 h at 37 °C, 600 rpm. Afterwards, wells were washed with PBS with 0.05% Triton × 100, and 0, 100 or 500 mM NaCl, and incubated with TMB substrate (Xema–Medica) for 10 min in dark and stopped with 100 µl of 10% HCl solution. The optical density was measured with Multiscan FC (Thermo Fisher Scientific) at 450 nm. Normalization of data was performed using subtraction of negative control optical density.

Antibacterial activity was assayed as mentioned above after the 3 h incubation of mixtures of endolysin (5 μg) and peptidoglycan in 5:1, 1:1 and 1:5 LysECD7-SMAP:LPS ratios.

### DNA binding assay

Bacterial DNA was isolated from an overnight culture of *A. baumannii* 50–16 strain with DNeasy UltraClean Microbial Kit (QIAGEN). DNA concentration was measured with Qubit DNA HS Assay Kit (Thermo Fisher Scientific) on Qubit 3.0 fluorimeter.

Endolysin (280 ng) was incubated with DNA in 10:1 and 2:1 LysECD7-SMAP:DNA ratio at room temperature for 30 min. Then mixtures were analyzed by electrophoresis in 1% agarose gel. Electrostatic interactions effect was assessed by adding 0, 50, and 150 mM NaCl to the mixtures during the incubation. Also, antibacterial activity of LysECD7-SMAP (1 μg ml^−1^) in the presence of DNA was assessed after incubation for 30 min as described above using in vitro activity test with *A. baumannii* 50–16 strain.

### Safety assessment

The hemolytic activity of endolysins was determined against human red blood cells (RBC). Fresh human blood was obtained in a heparin-containing tube and was centrifuged at 500 × *g* for 10 min, 4 °C. The pellet was washed three times with PBS buffer, and a solution of 10% RBC in PBS was prepared. RBC solution was mixed in a ratio of 1:1 with endolysins solutions in concentration of 100 µg ml^−1^ and 1 mg ml^−1^ in 20 mM Tris–HCl buffer (pH 7.5). PBS buffer was used as a negative control and 0.1% Triton X-100 was used as a positive control. The reaction mixtures were incubated for 1 h at 37 °C, harvested by centrifugation (500 × g, 10 min, 4 °C) and the absorbance at the wavelength of 405 nm of the supernatants was measured. All experiments were performed in triplicate, and the hemolysis rate was estimated as follows: Hemolysis (%) = (OD_exp_—OD_PBS_)/(OD_Triton_—OD_PBS_) × 100%, where OD_exp_ is OD_405_ in the experimental mixtures, OD_PBS_ is OD_405_ in the negative control (PBS) mixtures, and OD_Triton_ is OD_405_ in the positive control (0.1% Triton × − 100) mixtures.

### Cytotoxicity assays

Cytotoxicity was measured for HEK293 (ATCC-CRL-1573) cells by MTT assay. Cells were seeded at 4.5 × 10^4^ cells per well in 96-well plates and cultivated in 100 µl of DMEM medium supplemented with 10% fetal bovine serum, 2 mM glutamine, 50 U ml^−1^ penicillin and 50 µg ml^−1^ streptomycin for 24 h in a humidified atmosphere of 5% CO_2_ and 95% air at 37 °C. Afterwards, the medium was removed and 50 µl of endolysins serial dilutions in DMEM (from 1000 µg ml^−1^ to 16 µg ml^−1^) were added into each well. DMEM was used as a negative control and 0.1% Triton × − 100 was used as a positive control. Mixtures were incubated for 1 h (37 °C, 5% CO_2_) and the cells were then stained with 10% MTT solution in DMEM (final stain concentration is 0.5 µg ml^−1^) for 4 h (37 °C, 5% CO_2_). After incubation, the wells content was replaced by 100 µl of DMSO and the absorbance of the solutions at the wavelength of 570 nm was measured using SPECTROstar NANO (BMG LABTECH). All experiments were performed in triplicate, and the survival of HEK293 cells was estimated as follows: Survival (%) = (OD_exp_—OD_min_)/(OD_max_—OD_min_) × 100%, where OD_exp_ is OD_570_ in the experimental culture wells, OD_min_ is OD_570_ in the positive control (0.1% Triton ×− 100) culture wells, and OD_max_ is OD_570_ in the negative control (DMEM) culture wells.

Alternatively, HEK293 cells were seeded into 24-wells plates in completed DMEM (Gibco), supplemented with 1% Antibiotic/Antimycotic solution (Capricorn) and 10% fetal bovine serum (HyClone), the day before the experiment. Then, the cells were treated with different concentrations of LysECD7-SMAP and incubated at 37 °C and 5% CO_2_. After 48 h of incubation, the cells monolayer was fixed with 5% formaldehyde solutions and stained with 0.5% crystal violet (Sigma) and cells disaggregation was assessed.

### Determination of bacterial resistance

Resistance development was tested using the repeated exposures of endolysins on bacterial cultures in plate lysis assay and antibacterial assay on initial and passed strains of *A. baumannii* 50–16 and *K. pneumoniae* Ts 104–14. Overnight bacterial cultures were diluted in LB broth and grown at 37 °C, 250 rpm to an exponential phase (OD_600_ = 0.6). Subsequently, 200 µl of the bacterial suspension was spread over a Petri dish with LB agar and air-dried at room temperature. Then, 10 µl of 1 mg ml^−1^ endolysins solutions or control (20 mM Tris–HCl buffer, pH 7.5) were dropped onto the bacterial lawn, and the dish was treated for 16–18 h at 37 °C. Next day, bacteria from the sub-lethal lysis zone with a not fully cleared lawn were scraped and incubated in LB broth to exponential phase at 37 °C with constant shaking to generate a new bacterial lawn for the next passage. Ten passages were done for *A.* *baumannii* and *K. pneumoniae*. Cultures from initial and passed strains were used to assess antibacterial activity of endolysins as described before in 50 µg ml^−1^ concentration. All experiments were performed in triplicate.

### Endolysin dosage forms formulation

To obtain dosage forms for parenteral administration, 20 mg ml^−1^ sterile LysECD7-SMAP solution was diluted with sterile PBS pH 7.4 to final concentrations of 2.5 mg ml^−1^ or 5 mg ml^−1^, supplied with 0.01% Poloxamer 188 and freeze-dried using FreeZone 2.5 Liter Benchtop Freeze Dryer (Labconco) resulting in lyophilized powder for injections. Lyophilized powders were dissolved in deionized water immediately before the experiment.

Gel preparations for topical administration were formulated with LysECD7-SMAP, containing 5 mg ml^−1^ or 10 mg ml^−1^ of protein, 1% Natrosol 250 HHX (w/w) and 1.5% PEG 1500 (w/w). The gel base was mixed separately and sterilized at 120 °C for 15 min. Further sterilized endolysin solution in appropriate concentration was added to the gel and mixed thoroughly. Placebo gel was prepared the same way using PBS solution instead of active compound.

### Animal models

All animals were housed in separate cages with controlled temperature (20–24 °C) and humidity (45–65%), fed with a balanced rodent diet and water ad libitum.

### Murine sepsis model

Four-week-old female BALB/c mice (weight 16–18 g) were intraperitoneally inoculated with 200 µl of *K. pneumoniae* M9 suspension in PBS. Based on toxicological study results the sepsis infection in mice was established by inoculating 1 × 10^3^ CFU per animal of *K. pneumoniae* M9. At 2 h after infection mice were intravenously treated with 100 µl of LysECD7-SMAP solutions at protein concentrations of 2.5 mg ml^−1^ (n = 33) or 5 mg/ml (n = 33). For 15 animals the survival analysis was conducted and 6 animals were euthanized to assess the bacterial loads at each time point (1st before the beginning of treatment, 3rd and 7th days post infection, PI). Control groups included mice treated with 100 µl of PBS pH 7.4 (placebo group, n = 33), 100 µl of gentamycin 40 mg ml^−1^ (positive control, n = 24) or 100 µl of ampicillin 100 mg ml^−1^ (negative control, n = 24), in antibiotic groups 3 mice were euthanized for bacterial assessment at each time point. Also 33 mice were left untreated. The animals were treated twice a day with 10 h interval for 5 days.

To access the endolysin therapeutic effect general examination of animals, body weight and survival rate were observed daily for 8 days after the infection. Also, microbial contamination of spleen and lungs homogenates (on the 1st, 3rd and 7th days PI) and blood samples (on the 1st and 3rd days) were assessed by inoculation of blood or organ homogenates dilutions on GRM-1 agar medium. CFU count of *K. pneumoniae* was estimated after plates cultivation for 18 h at 37 ºC.

### Murine pneumonia model

Four-week-old female BALB/c mice (weight 16–18 g) were intranasally inoculated with 20 µl of *K. pneumoniae* M9 suspension in PBS (2 × 10^6^ CFU per animal). At 2 h after infection mice were intravenously treated with 100 µL of LysECD7-SMAP solutions at protein concentrations of 2.5 mg ml^−1^ (n = 24) or 5 mg ml^−1^ (n = 24). Control groups included mice treated with 100 µl of PBS (placebo group, n = 24) or 100 µl of gentamycin 40 mg ml^−1^ (positive control, n = 24). Also 24 mice were left untreated. In this model we did not used ampicillin control group as it showed low effectiveness in the sepsis in combating the infectious agent comparable to the level of untreated animals. In each group, for 15 animals the survival analysis was conducted and 3 animals were euthanized to assess the bacterial loads at each time point (1st before the beginning of treatment, 3rd and 7th days, PI). The animals were treated twice a day with 10 h interval for 5 days.

To access the endolysin therapeutic effect general examination of animals, body weight and survival rate were observed daily for 8 days after infection. Also, microbial contamination of spleen and lungs homogenates (on the 1st, 3rd and 7th days PI) and blood samples (on the 1st and 3rd days) were assessed by inoculation of blood or organ homogenates dilutions on GRM-1 agar medium. CFU count of *K.* *pneumoniae* was estimated after plates cultivation for 18 h at 37 ºC.

### Diabetic wound infection

In the diabetic wound model 19 male leptin-deficient db/db mice (BKS.Cg-Dock7m + / + Leprdb/J, Wildtype for Dock7m, Homozygous for Leprdb, The Jackson Laboratory) were maintained until their blood glucose (non-fasted) concentration reached at least 15 mmol l^−1^ (≥ 270 mg dl^−1^) or higher [32] for two days. The blood was collected from a warmed tail vein and analyzed by glucose analyzer (OneTouch Select® Plus Flex, LifeScan Johnson&Johnson). The age of mice at start of experimental time frame was 9 weeks.

When type 2 diabetes mellitus developed, as indicated with the blood test, the mice backs were shaved with an electric razor and four full-thickness skin wounds (d = 4 mm) were made with biopsy gun Dermo-Punch (Sterylab, Italy). Each wound was used to study either wound swabs contamination, either dermal graft homogenates contamination, either wound-closure course or histological examination.

Five minutes later each wound was infected with 1.5 × 10^8^ CFU per animal of *P. aeruginosa* 38–16 clinical isolate (25 µl in PBS). Mice were randomly allocated into experimental and control groups and at 24 h PI, the infected wounds were epicutaneously treated with 50 µl of LysECD7-SMAP gel at protein concentration 10 mg ml^−1^ (n = 8), placebo gel (n = 8) or left untreated (n = 3). The animals were treated twice a day with 10 h interval for 5 days.

To access the endolysins therapeutic effect the dynamics of wound healing, wound size and microbial contamination were observed on 1, 3, 7, 10 and 14 days after infection. CFU count of *P. aeruginosa* was estimated after the swabs of the wounds with sterile cotton balls wetted with saline were performed, serially diluted and plated on Agar B Medium. After 14th day of experiment, the mice (n = 4 in experimental groups and n = 3 in untreated group) were euthanized by CO_2_ inhalation, heart blood samples were taken and the infected dermal grafts were excised and homogenized in 1 ml of PBS. The solutions were serially diluted, plated on Pseudomonas Agar B Medium (for Fluorescein), containing peptone (20.0 g l^−1^), agar (15.0 g l^−1^), glycerol (10.0 g l^−1^), MgSO_4_·7H_2_O (1.5 g l^−1^), K_2_HPO_4_ (1.5 g l^−1^) and bacterial CFU were counted after an overnight incubation at 37 °C. Wound-closure course (digital planimetry) was analyzed by tracing the wound margin. For this, the pixel area of wound image was calculated using Adobe Photoshop CS6 software.

### Histology

One of four wounds in LysECD7-SMAP, placebo and untreated group was used for histological examination in the diabetic wound model. For mice euthanized on the 14th day dermal grafts were excised with biopsy gun (d = 8 mm, Sterylab) and fixed with 10% formaldehyde. Then biopsy samples were processed and embedded in paraffin (Leica TP 1020), and sectioned at 4 μm (MICROM HM340). These sections were deparaffinized, dehydrated, applied on glass slides and stained with hematoxylin/eosin according to the manufacturer’s instructions to assess wound healing processes. Tissue sections were analyzed with Leica DM LB2 microscope equipped with a Leica D300 digital camera (Leica).

### Bacteria genotyping

DNA was extracted from pure cultures using the QIAamp DNA Mini Kit (Qiagen) or RIBO-prep (Amplisens) as recommended by the manufacturers. To control for DNA contamination an additional sample containing MQ water was processed in parallel. DNA quantification were evaluated with Qubit 4 and Qubit dsDNA HS (Thermo Fisher Scientific, USA). 16S rRNA genes were amplified using Phusion High Fidelity DNA Polymerase (NEB, USA) and primer pair 27F and 1492R [33] according to the manufacturer’s instructions. PCR product purification was conducted with Cleanup S-Cap (Evrogen). The DNA sequencing was performed with the BigDye Terminator v3.1 Cycle Sequencing Kit (Applied Biosystems, USA) following manufacturer instruction. The reaction with BigDye v3.1 was purification using xTerminator® BigDye Purification kit (Applied Biosystems, USA) and the sequencing product was by the 3500 Genetic Analyzer (Thermo Fisher Scientific, USA). Sequencing analyses were performed using DNA Baser Sequence Assembler v.5.15 (Heracle BioSoft S.R.L.). Taxonomic identification was performed using BLAST*.*

### Burn wound infection

In the burn wound model induced with *P. aeruginosa* 38–16 clinical isolate (sputum of ICU patient), backs of 8–10 weeks old male Wistar rats (180–210 g) were shaved with an electric razor. To induce skin burn, a copper plate (surface area 150 mm^2^) was heated to 300 °C and then applied on the shaved backs of the rats for 30 s. To restore the water-electrolyte balance 1 ml of PBS buffer was injected subcutaneously to the burn areas. Five minutes later the burn was infected with 2 × 10^9^ CFU per animal of *P. aeruginosa* (100 µl in PBS). *P. aeruginosa* 38–16 shows resistance towards ampicillin; chloramphenicol; ceftazidime; cefotaxime; gentamicin; meropenem; polymyxin and tetracycline, moderate resistance to polymyxin B, as estimated by MIC assay microdilution method in Mueller–Hinton broth (Difco) [34]. The strain was not lethal in animal models when applied epicutaneously, however, in the absence of treatment, a stable infection develops with the preservation of wounds contamination up to 3 weeks.

At 24 h PI, the infected burns were epicutaneously treated with 500 µl of LysECD7-SMAP gel at protein concentrations of 5 mg ml^−1^ (n = 7) or 10 mg ml^−1^ (n = 7). Control groups included rats treated with 500 µl of vehicle-treated animals (placebo group, n = 7). Also 7 animals were left untreated. The animals were treated twice a day with 10 h interval for 5 days.

To assess the endolysins therapeutic effect the dynamics of burn wound healing, wound size and microbial contamination were observed on 1, 3 and 7 days after infection. CFU count of *P. aeruginosa* was estimated after the swabs of the wounds with sterile cotton balls wetted with saline were performed, serially diluted and plated on Pseudomonas Agar B Medium. After 7 days of experiment, the rats were sacrificed by CO_2_ inhalation, and the infected dermal grafts and animal spleens were excised and homogenized in 1 ml of PBS. The solutions were serially diluted, plated on Agar B Medium and bacterial CFU were counted after an overnight incubation at 37 °C. The wound-closure rate during the treatment course was monitored with the planimetry.

### Statistical analysis

Data were analysed using GraphPad Prism 9.0 software. A value of p < 0.05 was considered statistically significant. The methods used for the comparative tests are indicated in the captions of the figures and tables.

## Results

### Structural analysis indicates endopeptidase activity of LysECD7 and its SMAP fusion

**Fig. 1:**
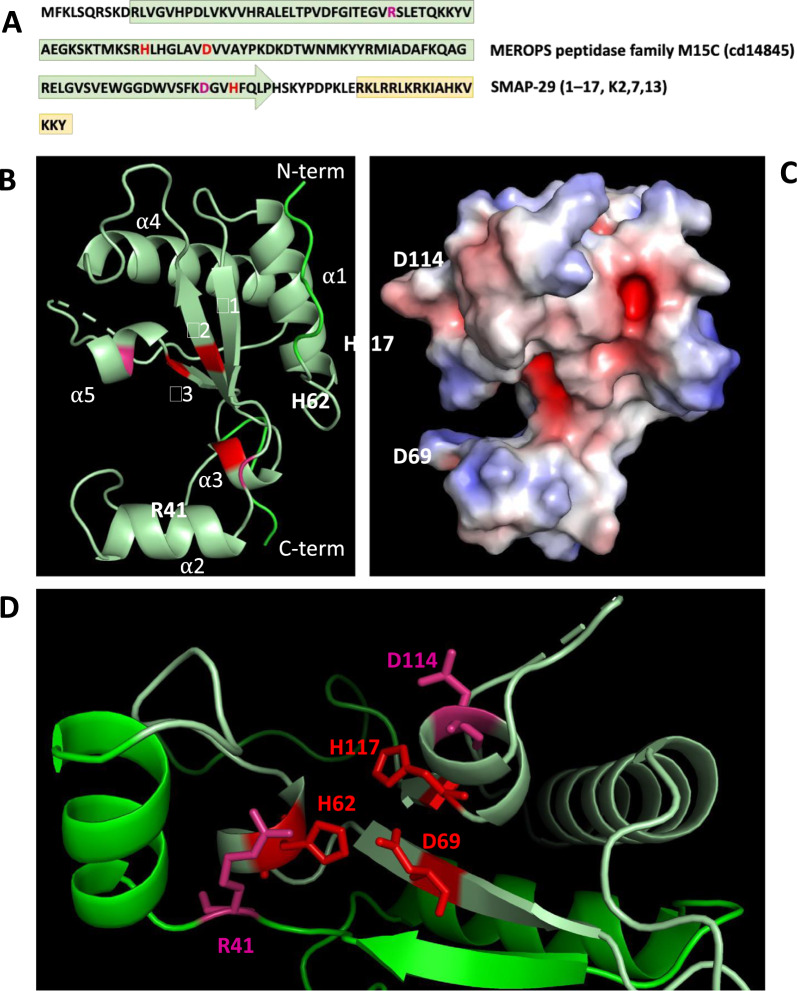
LysECD7 structural organization. **A** Primary amino acid sequence with predicted functional domains. Amino acids forming the active center of the peptidase are colored with red (Zn-binding aa) or magenta (charged aa) colors. SMAP in the LysECD7-SMAP fusion is highlighted in yellow. **B** 3D structure of LysECD7. Pale green indicates the location of Peptidase M15C domain. **C** Electrostatic potentials of LysECD7 surface. Negatively charged surface is colored red, positively charged surface is colored blue. **D** Active center of the LysECD7, the Zn^2+^ is coordinated with H62, D69 and H117 in the center of a shallow groove that is proposed to be the PG-binding pocket

The LysECD7 is predicted to be a zinc-binding metallopeptidase with L-alanyl-D-glutamate endopeptidase activity (cd14845) cleaving the peptidoglycan (PG) stem peptide. As вeduced from the 3D structure deposited under PDB ID 8Q2G, its domain architecture shares the conserved structure of endopeptidases of the MEROPS peptidase M15 family with an antiparallel, three-stranded β-sheet bordered by five α-helixes [35, 36] (Fig. 1A, Supplementary material). The active center of the enzyme contains three amino acids coordinating Zn^2+^ ion (H62, D69 and H117) and two additional charged acids (R41 and D114) bound to the water molecule for Zn^2+^ coordination, forming a charged groove important for PG binding [35, 37]. LysECD7 also contains the SxHxxGxAxD motif (60–69 aa) in the cleft region conserved for LAS-type enzymes [36].

For endolysins acting against Gram-negative bacteria, the presence of an additional membrane-translocating domain is predicted, allowing the enzymes to cross the outer membrane of bacteria and reach the peptidoglycan substrate [38, 39]. There is no obvious characteristic sequence on the ether C- or N-terminus of LysECD7, but a region enriched in charged and polar amino acids is detected in the α2 helix around 44–61 aa of the chain, predicted to be intrinsically-disorder region (IDR) with strong polyampholytic properties (FCR = 0.44) and low overall hydrophobicity (2.76), also containing a putative antimicrobial peptide. It forms a protrusion covering the substrate binding pocket of the enzyme and is enriched in lysine residues along the inner surface. Such elements can lack a compact ordered structure, e.g., under conditions of neutral pH in vitro, while under certain conditions the structure is stabilized and the domain acquires functionality [40], either the IDR can fold upon binding to interaction partners such as PG and other bacterial cell membrane structures. An additional text file describes this in more detail (see Additional file 2).

LysECD7 reveals a wide spectrum of activity against Gram-negative bacterial species, but it is not stable enough in PBS and human blood sera [16, 41]. Thus, this molecule was modified with the N-terminal α-helix of SMAP cationic peptide to enhance the antimicrobial properties, suggesting SMAP-mediated permeabilizing activity [42, 43] of the fusion. Comparison of the solved structures of native and hybrid molecules revealed only a slight difference in two α-helices involving α2 and α5, which together with the β1-β3 sheet form peptidoglycan-binding pocket (Additional file 2), resulting in a more closed cleft architecture of LysECD7-SMAP. We could not resolve the SMAP peptide structure, due to its high mobility, thus the LysECD7-SMAP model was used to perform molecular dynamics simulations. During the simulation the ‘core’ LysECD7 part remains stable (residues 1–131), while the structure of SMAP fragment is remarkably variable (residues 132–148), proposing the free interaction with bacterial membranes. Both domains appear to behave independently, as SMAP tends to be located away from the ‘core’ globule. Apparently, the fusion of two domains into a chimeric polypeptide does not significantly alter the properties of these domains, preserving the functionality of each (Additional file 2).

### LysECD7-SMAP active against a broad spectrum of *bacteria* and bacterial biofilms

**Fig. 2:**
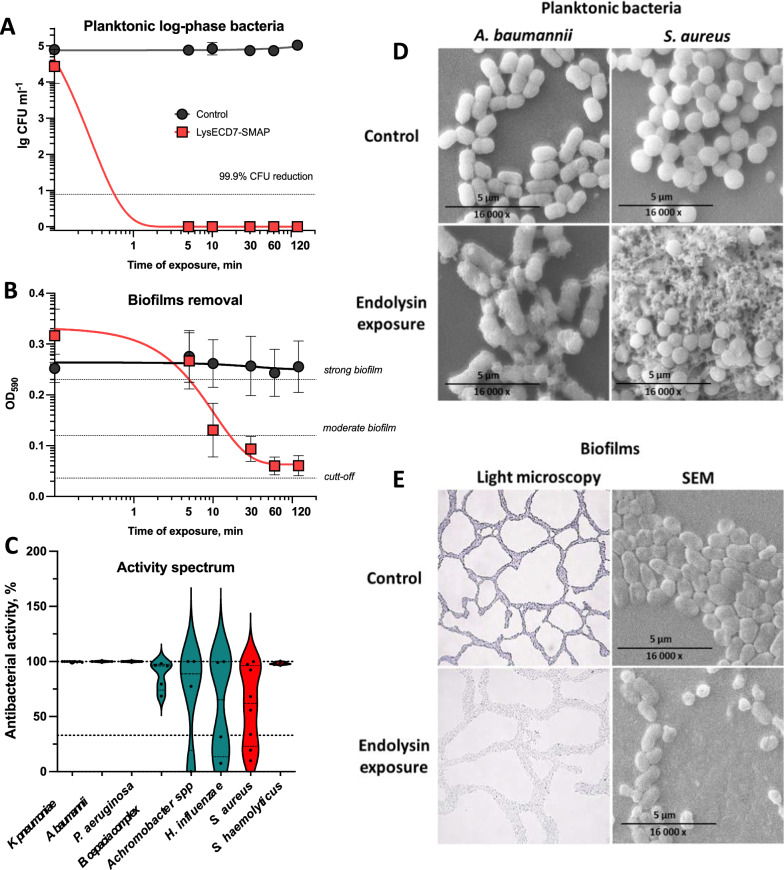
LysECD7-SMAP in vitro antibacterial activity. **A** Time-kill curve of LysECD7-SMAP at a concentration of 100 μg ml^−1^ of logarithmic-phase growing planktonic *A. baumannii* 50–16 cells. **B** Time-kill curve of LysECD7-SMAP at a concentration of 100 μg ml^−1^ of *A. baumannii* 50–16 formed biofilms. Data are shown as mean ± standard error of mean (SEM) (one-way ANOVA). **C** LysECD7-SMAP spectrum of action (100 µg ml^−1^ protein concentration). Violin plot; dots indicate activity towards individual strains; lines, median activities for the species (G- in green, G + in red). The 33% activity cut-off is indicated with a dotted line. **D** Scanning electron microscopy images of the LysECD7-SMAP bactericidal activity against *A. baumannii* 50–16 and *S. aureus* Z 73–14 planktonic cells (10 µg ml^−1^, 30 min exposure). **E** Light and scanning electron microphotographs of the LysECD7-SMAP antibiofilm activity against *A. baumannii* 50–16 biofilms formed on glass slides (100 µg ml^−1^, 2 h exposure)

To investigate the potential of LysECD7-SMAP as an antibacterial agent, we evaluated its in vitro activity against planktonic cells and preformed biofilms (BF). We observed complete elimination of exponentially growing planktonic *A. baumannii* 50–16 after 5 min of exposure at a protein concentration of 100 µg ml^−1^ (Fig. 2A). When assessing antibiofilm activity, disruption of preformed biofilms was observed after 30 min of exposure, and almost complete elimination of BF (up to 77.6% BF reduction) occurred after 1 h (Fig. 2B).

The antibacterial activity spectrum of LysECD7-SMAP screening included clinical isolates of nosocomial pathogens from both Gram-negative and Gram-positive bacterial species (Fig. 2C). Endolysin was active against all isolates of *K. pneumoniae, A. baumannii, P. aeruginosa* and *S. haemolyticus* tested*,* with median antibacterial activity of 98–100%. LysECD7-SMAP was also active against *Achromobacter spp, B. cepacia complex* and* H. influenzae,* as well as *S. aureus,* but not all the strains were susceptible. Both LysECD7-SMAP and LysECD7 showed 100% antibacterial activity against two strains of *Bacillus subtilis* tested. Considering that unmodified LysECD7 lacks activity against other Gram-positive bacteria, e.g. *S. aureus* [16], we propose the substrate specificity towards A1γ-type peptidoglycan stem peptides specific for *Bacillus* spp. and most Gram-negative species.

**Fig. 3:**
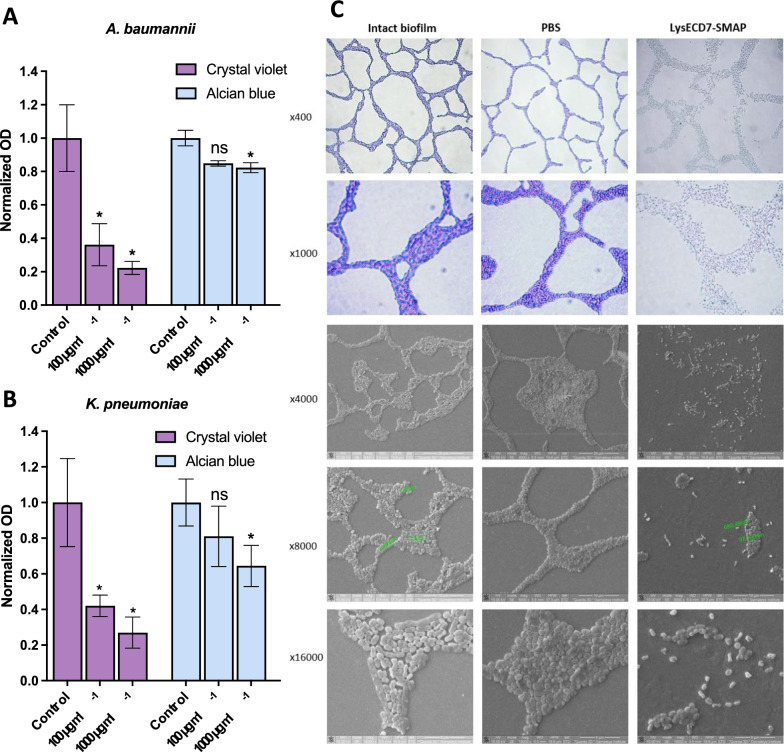
LysECD7-SMAP activity against bacterial biofilms. **A** Antibiofilm activity of LysECD7-SMAP against *A. baumannii* formed biofilms stained with crystal violet and alcian blue. OD_590_ and OD_615_ were measured after 2 h of exposure to endolysin. **B** Antibiofilm activity of LysECD7-SMAP against *K. pneumoniae* formed biofilms stained with crystal violet and alcian blue. OD_590_ and OD_615_ were measured after 2 h of exposure to endolysin. Data are shown as mean normalized to mean control values ± SD (two-way ANOVA). *p < 0.05, **p < 0.005, ***p < 0.001. **C** Microscopy of *A. baumannii* bacterial films under action of endolysin at concentration 1 mg ml^−1^

Electron microscopy confirmed the bactericidal activity of LysECD7-SMAP against Gram-negative and Gram-positive bacteria. Completely disrupted or significantly crumpled bacterial cells were detected in the debris aggregates after incubation with LysECD7-SMAP (Fig. 2D), indicating direct interaction with bacterial cell wall components. Loss of biofilm integrity and degradation of the extracellular polymeric substance (EPS) matrix was also observed (Figs. 2E, 3). BFs after endolysin exposure were stained with non-specific crystal violet dye or specific Alcian blue dye, whicht stains biofilm polysaccharides [44]. LysECD7-SMAP significantly disrupted biofilms with low (*A. baumannii*) and high (*K. pneumoniae*) polyanionic PS/PNAG content in a dose-dependent manner (Fig. 3). While the specific catalytic interactions with a variety of exopolysaccharides are unlikely, the electrostatic binding of endolysins with acidic EPS polysaccharides is quite possible, contributing to detachment and disruption of the biofilms structure.

### Interactions of LysECD7-SMAP with specific molecular targets influence its activity

The antimicrobial activity of LysECD7-SMAP is mediated by the catalytic activity of the endopeptidase LysECD7 and the permeabilizing effect induced by the SMAP peptide. The SMAP sequence represents a cationic peptide capable of interacting with the charged cell walls and BF targets such as lipopolysaccharides, biofilm extracellular DNA, etc., interfering its antimicrobial action.

We have shown that increasing the level of PG in samples incubated with endolysin can significantly reduce LysECD7-SMAP activity, accompanied by the formation of a high molecular weight product visible by electrophoretic activity shift assay (Additional file 3, Fig. S3a, b). Apparently, the irreversible PG interaction explains the decrease in enzyme activity and the appearance of bacterial cell aggregates embedded in cell debris visible by SEM. As a result, the trapped, aggregated lysin molecules were no longer able to attack the bacterial cells in suspension. Purified LPS also affects LysECD7-SMAP bactericidal activity (Fig. S3c, d): complete inhibition of its activity was observed at ratio 1:1 (m/m). However, no visible changes in protein profile were detected. The loss of activity could be due to non-specific interactions between positively charged endolysin fragments and negatively charged LPS, consistent with the ELISA data (Fig. S3e). We propose that the bactericidal activity of LysECD7-SMAP may also be associated with the membrane destabilizing activity due to LPS binding, resulting in the loss of LPS-induced bacterial membrane effects, allowing the protein to reach the PG [45, 46]. Apparently, the hybrid protein is also able to act on strains with modified LPS substitutions, with the lower negative charge and a stronger permeability barrier, destabilizing a more tightly packed LPS layer. LysECD7-SMAP also interacts with bacterial DNA, followed by loss of enzyme activity in a non-specific electrostatic manner, as shown by addition of NaCl (Fig. S3f-h). These data suggest that after cell lysis, binding to released compounds such as polysaccharides or nucleic acids may be the pathway for enzyme inactivation.

### LysECD7-SMAP formulations effective in systemic infection models

Positive results from in vitro experiments encouraged us to test the therapeutic potential of LysECD7-SMAP in vivo in two systemic and two topical infectious animal models (Additional file 4, Fig. S4a). In developing the systemic and topical formulations, various excipient and pH options were considered, with the most stable and active formulations selected for further study (PBS pH 7.4 supplied with 0.01% Poloxamer 188 for lyophilized powder for injections and Natrosol-based gel pH 7.4). Long-term stability testing was performed on three lyophilizate and gel batches at 5 °C ± 3 °C and 60 ± 5% relative humidity, monitoring antibacterial activity, identification tests, pH and sterility. The gel dosage form was shown to meet all acceptance criteria for 6 months, while the lyophilizate was stable for at least 1 year.

**Fig. 4:**
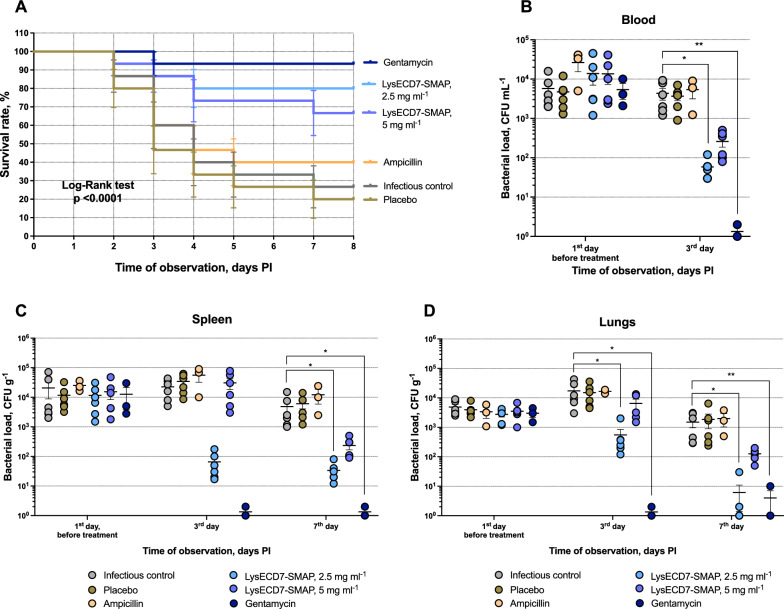
Therapeutic efficacy of LysECD7-SMAP in a *K. pneumoniae* sepsis model. **A** Survival rates of the murine model (n = 15). The log-rank (Mantel-Cox) test was used for analysis. **B-D** Bacterial loads in the blood (b), spleen (c) and lungs (d) during the treatment course, n = 6 at each time point for all groups except antibiotics (n = 3). Data are shown as the mean ± SEM. Asterisks indicate a significant difference in CFU count compared to the untreated control (Kruskal–Wallis test, Dunn’s multiple comparisons test). *p < 0.05, **p < 0.005

For the *Klebsiella* sepsis model, infection was induced in mice by intraperitoneal injection of *K. pneumoniae* M9, which is characterized by high virulence, resistance to beta-lactams (ampicillin, MIC ≥ 32 mg l^−1^) and susceptibility to gentamicin (MIC = 0.25 mg l^−1^). Preparations with two protein concentrations were assessed 2.5 mg ml^−1^ or 5 mg ml^−1^. The survival rate was assessed for 8 days (Fig. 4A). By the end of the observation, the survival rates in untreated control and placebo groups were 26.6 and 20%. Survival in the antibiotics groups was 40.0% for ampicillin and 93.3% for gentamicin, the latter being the highest of all the groups studied. Survival was significantly higher in the LysECD7-SMAP-treated groups compared to infectious control (IC) or placebo animals: 80.0 and 66.7% for 2.5 and 5 mg ml^−1^ solutions, respectively. A decrease in animal weight was shown in untreated and placebo-treated animals at 8 days post infection (PI). However, necropsy of the animals didn’t reveal any organ abnormalities except for an enlarged spleen.

A decrease in CFU counts in organs and blood was observed in gentamycin and LysECD7-SMAP, 2.5 mg ml^−1^ groups compared to IC (Fig. 4B, C, D). Decolonization in the endolysin group in lung and spleen achieved median reductions of 2.9 and 2.0 lg CFU g^−1^ of organ (94.9% and 64.1% of the initial bacterial loads in the group) and 1.8 lg CFU ml^−1^ (55.9% of the initial load) in blood. Thus, the lung bacterial load in the in LysECD7-SMAP, 2.5 mg ml^−1^ group was equivalent to the CFU levels in the gentamicin group two days after the end of treatment (7th day). The effect of LysECD7-SMAP at a protein concentration 5 mg ml^−1^ was less pronounced, compared to 2.5 mg ml^−1^.

**Fig. 5:**
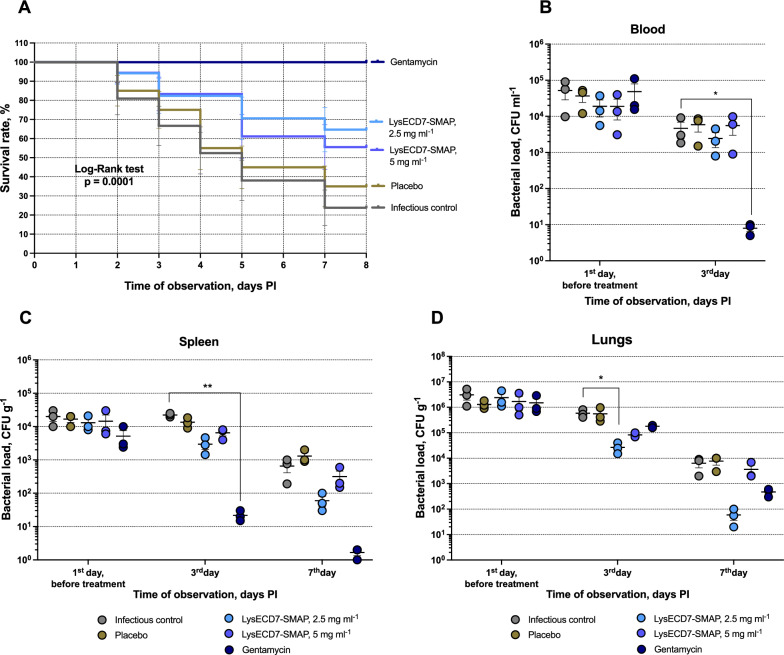
Therapeutic efficacy of LysECD7-SMAP in a *K. pneumoniae* pneumonia model. **A** Survival plot (n = 15). The log-rank (Mantel-Cox) test was used for analysis. **B-D,** Bacterial loads in the blood (b), spleen (c) and lungs (d) during the treatment course, n = 3 at each time point. Data are shown as the mean SEM. Asterisks indicate a significant difference in CFU count compared to the untreated control (Kruskal–Wallis test, Dunn’s multiple comparisons test). *p < 0.05, **p < 0.005

In the pneumonia model, infection was induced by intranasal inoculation with *K. pneumoniae* M9. By the end of follow-up, we observed the maximum survival rate in the gentamicin (100%) and LysECD7-SMAP, 2.5 mg ml^−1^ (64.7%) groups, whereas almost three times more animals died in the IC group (survival rate 23.8%) (Fig. 5A).

Tenfold treatment of animals with LysECD7-SMAP, 2.5 mg ml^−1^ also eliminated bacterial contamination in the lungs by 2.2 lg CFU g^−1^ of organ on day 7 PI compared to the control and resulted in a 71.8% reduction from the initial load (Fig. 5D). No significant antibacterial effect was observed for LysECD7-SMAP, 5 mg ml^−1^. It is worth noting that in the gentamicin group, the effect in the lungs was less pronounced than the effect of endolysin treatment. However, there was no significant decolonization in the spleen or blood samples of the experimental groups throughout the follow-up period (Fig. 5B, C).

### Treatment of diabetes-associated wound infection with LysECD7-SMAP

The efficacy of the lysin-based antimicrobial gel treatment was investigated in impaired diabetic ulcer healing in leptin receptor-deficient mice. The clinical isolate *P. aeruginosa* 38–16 was used to model local wound infection. To determine the effects of the gel, wound closure rate and bacterial decolonization were assessed during the course of treatment. Prior to injury and infection of the mice, the development of trophic disorders associated with diabetes was confirmed to verify the model (Additional file 4).

**Fig. 6:**
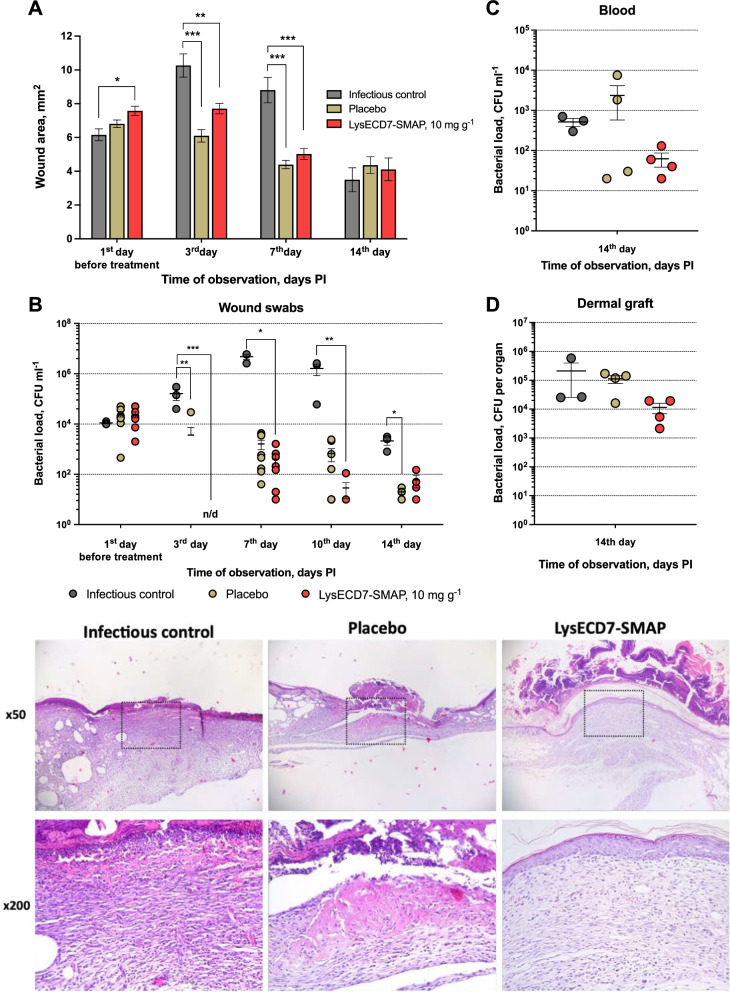
LysECD7-SMAP gel application in diabetic wound infection model caused by *P. aeruginosa* 38–16. **A** Course of wound closure in the studied groups. **B-D** Bacterial loads of wound swabs (B), blood (C) and dermal graft homogenates (D) during the treatment course. Data are shown as the mean ± SEM. Significant differences are shown as asterisks, otherwise no statistical difference is found (Kruskal–Wallis test, Dunn’s multiple comparisons test), *p < 0.05, **p < 0.005, ***p < 0.001. **E** Histological re-epithelialization of dorsal skin wounds at 14th day PI. Paraffin sections of tissues from db/db mice were stained with hematoxylin and eosin. Dorsal wound closure was monitored for each animal and representative photographs of three groups at original magnifications × 50 (upper) and × 200 (lower) are shown. The enlarged regions are defined with dashed squares

Before the start of treatment (1st day, 24 h PI), the open wound area was 6–8 mm^2^ depending on the group (Fig. 6A). On day 3 PI and during the withdrawal period (day 7 PI), both placebo and LysECD7-SMAP gel significantly (p < 0.005) accelerated wound healing in mice compared to IC. At a later stage (day 14), tissue repair begins in the infectious control group and no statistically significant differences were found between the study groups. Although the animals' wounds showed similar wound closure rates in all groups two weeks after skin injury, there were differences in the wound histology of LysECD7-SMAP and placebo-treated animals compared to the untreated group (Fig. 6E). By day 14, complete re-epithelialization was observed in 100% of biopsy sections in LysECD7-SMAP-treated animals and 75% in placebo-treated animals, whereas in untreated animals re-epithelialisation was completely absent and an extensive ulcerative defect covered with necrotic debris was observed.

When assessing the bacterial load of skin swabs, a significant reduction in CFU was detected in the LysECD7-SMAP and placebo groups compared to untreated animals (Fig. 6B). Application of LysECD7-SMAP significantly reduced (p < 0.005 and 0.001) the bacterial load of swabs during treatment (day 3) and up to day 10 PI, reaching a reduction of 5.3–4.5 lg units. In the placebo group significant (p < 0.05) decrease was detected on the 3rd and 14th days PI by 1.7 and 2.0 lg units. This confirms the efficacy of the developed dosage form, as the antibacterial effect was maintained nine days after the end of gel administration. Bacterial contamination of skin graft homogenates at the wound surface and bacterial load of blood samples (Fig. 6C and D) at day 14 PI showed no differences in efficacy of the lysin gel or vehicle-treated groups compared to IC. However, the median bacterial load in the LySECD7-SMAP group was reduced by 1.04 and 0.33 CFU ml^−1^ in blood or dermal graft homogenates compared to IC.

In the local infection model, two parallel processes occur—pathogen elimination and tissue repair. The gel excipients (Natrosol 250 HHX and PEG) have been selected to enhance the effect of endolysin and improve wound healing properties. Thus, in the chronic wound treatment, the placebo gel itself has an effect on wound healing, but not as pronounced as in the case of the LysECD7-SMAP gel, which is particularly evident in histological observations. Insignificantly faster wound healing in the vehicle group is not confirmed by cultural or histological examination and can be explained by bacterial lysis in the presence of lysin, with the release of cellular components provoking immune responses around the wound defect. In addition, the microbiological composition of wound swabs, dermal grafts and blood samples differed between groups (Additional file 4, Table S3), with a predominance of target *P. aeruginosa* only in the IC. Combining the data on bacterial loads and colony genotyping, we expect an almost sterilizing reduction of target *P. aeruginosa* cells on the wound surface by the third day of endolysin-based gel application, with strain preservation in the depth of the wound defect and moderate effects on the side wound flora (mainly Gram-negative strains). This is followed by wound colonization with commensal microorganisms pre-existing in the wound area, which however do not prevent skin re-epithelialization. The efficacy of topical treatment was also confirmed in the acute burn wound infection model described in detail in Additional file 4.

## Discussion

Peptidoglycan-hydrolyzing phage endolysins are in the trend of non-conventional therapies to combat drug-resistant infections [47]. Functional engineering of enzymes allows to prolong their half-life, improve biodistribution and maintain high activity in the bloodstream and tissues [5]. Modifications with different functional sequences, including fusion with translocation and binding domains, hydrophobic, polycationic or antimicrobial peptides, allow their action to be extended to more complex diseases and infectious agents [3, 4, 48]. This is particularly important for endolysins targeting Gram-negative bacteria, as the presence of an outer membrane has long hindered the development of endolysin-based drugs [49].

LysECD7-SMAP is a fusion of LysECD7 endopeptidase and a modified fragment of the polycationic peptide SMAP-29 [50], which has an improved in vitro and in vivo activity, compared to the unmodified protein. It was developed to obtain an optimized pharmaceutical ingredient [16] for topical the administration and treatment of systemic infections caused by a broad spectrum of both Gram-negative and some Gram-positive species that were not susceptible to a parent LysECD7. Here we have shown, that hybridization of two functional sequences (catalytic domain and AMP) does not affect their properties, allowing both to interact with bacterial membranes in an independent manner. The modified enzyme has significant destabilizing activity on planktonic cells and biofilms, achieved through both specific and non-specific interactions with components of a complex bacterial microenvironment. At the same time, it shows no activity against eukaryotic cells or signs of cytotoxicity, indicating no significant safety concerns (Additional file 5).

Various concentrations of LysECD7-SMAP have been formulated into parenteral injection forms and topical gel and studied in several animal models. The tenfold, twice daily injections of LysECD7-SMAP in concentration 2.5 mg ml^−1^ (250 µg per animal or 12.5 mg kg^−1^ at once) resulted in the significant animals’ lifespan increase and reduced bacterial burden in *Klebsiella* pneumonia and sepsis models. Lower efficacy of the drug in the pneumonia model suggests that the protein may have hindered penetration into infected lung areas when administered intravenously, and inhaled or combined administration is required to eliminate bacteria from the lungs and bloodstream. In both systemic models, higher doses of LysECD7-SMAP resulted in slightly reduced survival rates. We believe this is due to rapid bacterial lysis with local release of bacterial components (LPS, PG, nucleic acids) leading to excessive inflammation and development of septic reactions. For example, similar results were observed for LysSS endolysin at high systemic doses [13].

Our results are consistent with other engineered and unmodified endolysins active against Gram-negative pathogens in models of systemic infection, where doses of 250–1000 µg of endolysin significantly prolonged animal lifespan by 40–60% compared to controls [12–14]. However, the systemic efficacy in terms of therapeutic protein pharmacokinetics could be improved by combining the routes of administration. For example, the most pronounced effect in the pneumonia model was demonstrated when endolysin was administered by intranasal and intratracheal instillation [6].

In contrast to systemic infections, wound infections are often associated with the formation of polymicrobial biofilms [51], that impede the access of antimicrobial agents, requiring increased application of antimicrobials. The treatment of chronic bacterial wound infections with endolysins is still poorly described. In general, the use of lysin solutions is not informative and the formulation of endolysins in a suitable gel is required to moisturize the wound and promote excessive pus drainage for a prolonged and stable effect. In addition, impaired wound healing in diabetes-associated ulceration results in chronic wounds with dynamic microbiome profiles [52, 53]. Therefore, intensive infection management is required alongside wound healing to prevent adverse outcomes such as limb amputation or patient death [54].

In chronic diabetic wound infection, the application of LysECD7-SMAP gel enabled re-epithelialization to be achieved by day 14 PI in all biopsies compared to control and vehicle-treated groups where re-epithelialization was partially or completely absent. Although, the main wound healing effect is obviously related to the composition of the gel base, the antibacterial effect is due to the action of endolysin, also by reducing the bacterial load in the blood of the animals, and was maintained during (3rd day PI) and after the end of treatment (7th day PI and later). Application of the gel at a dose of 10 mg ml^−1^ of LysECD7-SMAP in an acute burn wound infection has a pronounced effect, allowing the burn area to be reduced by approximately 25% of the initial value by day 7 PI and wound burden to be reduced by approximately ¾ of the initial bacterial load. Several topical formulations have been described in the literature. These include an Aquaphor® gel with modified endolysin PaP1, which reduced the bacterial load twice after a single application to burn wounds [38], but no long-term studies have been performed. However, most effective gel formulations with endolysins have been developed to combat gram-positive pathogens, mainly staphylococcal infections.

Despite significant advances in understanding the mechanism of action of enzybiotics, we still have little understanding of how these enzymes work in vivo, which hinders translational research on such antimicrobials. Our results in animal models suggest that treatment with lysin-based preparations is most effective in the early stages of infection. Timely use of such antibacterials can be critical due to their PK characteristics [55], which have been shown to eliminate from the organism relatively quickly. In this regard, treatment practice can be improved by adapting treatment regimens to include longer or more intensive use during the first days after infection. For pharmaceutical development, these results allow to justify the regimen and dosage of the preparations in clinical settings.

Alternative formulation strategies for prolonged enzyme release, such as chemical and genetic modification, immobilization of proteins in nanoparticles, are also being actively investigated and may be beneficial in improving their PK and PD characteristics [56–58]. However, such strategies often affect the activity of the enzyme itself and must be carefully selected on a case-by-case basis.

## Conclusion

Based on the results obtained, we believe that antimicrobials based on the modified phage endolysin LysECD7-SMAP are safe and effective in the treatment of infections caused by Gram-negative pathogens. To date, in vitro and in vivo preclinical studies, including efficacy, PK assessment and safety, have been completed for the topical formulation at a concentration of 10 mg g^−1^, showing absence of toxic effects after single and multiple administrations [55]. The systemic dosage form has also shown acceptable safety profiles in toxicology, immunotoxicity and allergenicity studies after single and repeated i.v. administration in rodents and is in the final stages of toxicology studies. Protocols for the clinical trials of the dosage forms are under development. We believe that our results can inspire the research towards intensification of endolysin-based antibiotics application to reduce the burden of MDR and XDR microorganism in public healthcare.

### Supplementary Information

Additional File 1. pdf. Supplementary tables S1 and S2. Tables containing information on coding sequences and structural analysis of LysECD7 and LysECD7-SMAP.

Additional File 2. LysECD7 structural traits and characteristics.

Additional File 3. Supplementary figure S3. LysECD7-SMAP interactions with components of bacteria.

Additional File 4. Additional data of LysECD7-SMAP efficacy studies.

Additional File 5. LysECD7-SMAP *in vitro* toxicity tests.

## Data Availability

Atomic coordinates and structure factors of the two crystal structures of LysECD7 and LysECD7-SMAP have been deposited in Protein Data Bank (LysECD7 PDB ID code 8Q2G). The source data are available from the corresponding authors on a reasonable request.
