# Supplementary material for: Development of novel antimicrobials with engineered endolysin LysECD7-SMAP to combat Gram-negative bacterial infections
Source: J Biomed Sci. 2024 Jul 24;31:75. doi: 10.1186/s12929-024-01065-y (PMC11267749; doi:10.1186/s12929-024-01065-y)
Supplement: Supplementary file 1 — Additional File 1. pdf. Supplementary tables S1 and S2. Tables containing information on coding sequences and structural analysis of LysECD7 and LysECD7-SMAP. [file 12929_2024_1065_MOESM1_ESM.docx]

**Table S1.** Coding sequences and primers used in the study

|  | **5'-Sequence-3’** | Description |
| --- | --- | --- |
| Nucleic seq | ATGTTTAAATTATCTCAAAGAAGCAAAGATCGGCTTGTTGGTGTGCATCCTGATCTGGTAAAAGTCGTTCATCGTGCATTAGAACTGACTCCGGTCGATTTTGGTATTACGGAAGGTGTACGTAGTCTAGAAACGCAAAAGAAATATGTTGCGGAAGGTAAATCTAAGACTATGAAATCTCGTCATCTTCACGGTTTAGCGGTTGACGTTGTAGCGTATCCGAAAGATAAGGATACATGGAACATGAAATACTACCGCATGATCGCGGATGCATTTAAACAAGCTGGTCGAGAATTGGGTGTTTCTGTTGAATGGGGTGGGGATTGGGTATCGTTTAAAGACGGTGTTCATTTTCAGCTACCACACAGCAAATACCCAGATCCAAAACTCGAG | LysECD7 coding sequence |
|  | ATGTTTAAATTATCTCAAAGAAGCAAAGATCGGCTTGTTGGTGTGCATCCTGATCTGGTAAAAGTCGTTCATCGTGCATTAGAACTGACTCCGGTCGATTTTGGTATTACGGAAGGTGTACGTAGTCTAGAAACGCAAAAGAAATATGTTGCGGAAGGTAAATCTAAGACTATGAAATCTCGTCATCTTCACGGTTTAGCGGTTGACGTTGTAGCGTATCCGAAAGATAAGGATACATGGAACATGAAATACTACCGCATGATCGCGGATGCATTTAAACAAGCTGGTCGAGAATTGGGTGTTTCTGTTGAATGGGGTGGGGATTGGGTATCGTTTAAAGACGGTGTTCATTTTCAGCTACCACACAGCAAATACCCAGATCCAAAACTCGAG*AGGAAGCTTCGAAGACTGAAGAGGAAGATAGCACATAAGGTGAAGAAGTATTAA* | LysECD7-SMAP coding sequence |
| Protein seq | MFKLSQRSKDRLVGVHPDLVKVVHRALELTPVDFGITEGVRSLETQKKYVAEGKSKTMKSRHLHGLAVDVVAYPKDKDTWNMKYYRMIADAFKQAGRELGVSVEWGGDWVSFKDGVHFQLPHSKYPDPKLE | LysECD7 amino acid sequence |
|  | MFKLSQRSKDRLVGVHPDLVKVVHRALELTPVDFGITEGVRSLETQKKYVAEGKSKTMKSRHKHGLAVDVVAYPKDKDTWNMKYYRMIADAFKQAGRELGVSVEWGGDWVSFKDGVHFQLPHSKYPDPKLE*RKLRRLKRKIAHKVKKY* | LysECD7-SMAP amino acid sequence |
| Endolysin cloning | | |
| pET42vecF | 5’-CTCGAGCACCACCACCACCACCACCACCACTAA-3’ | Vector part |
| pET42vecR | 5’-CATATGTATATCTCCTTCTTAAAGTTAAACAAAAT-3’ |  |
| ECD7- SMAPF | AAGAAGGAGATATACATATGTTTAAATTATCTCAAAGAAGCA | LysECD7-SMAP ORF |
| ECD7- SMAPR | CTTATGTGCTATCTTCCTCTTCAGTCTTCGAAGCTTCCT |  |
| Bacteria genotyping | | |
| 27F | 5’-AGAGTTTGATCMTGGCTCAG-3’ | 16S rRNA |
| 1492R | 5’-GGTTACCTTGTTACGACTT-3’ |  |

**Table S2.** Enzymes crystal data collection and refinement statistics

| **X-ray diffraction data collection statistics** | | |
| --- | --- | --- |
|  | **LysECD7** | **LysECD7-SMAP** |
| Space group | C222 | *P*2_1_2_1_2_1_ |
| Cell parameters (Å,°) | 75.57 82.33 64.60,  90 90 90 | 48.66 58.03 196.63,  90 90 90 |
| Wavelength (Å) | 0.9184 | 0.9184 |
| Resolution (Å) | 42.21 - 2.49 | 47.28 - 1.7 |
| Number of reflections | 65130 | 432567 |
| Number of unique reflections | 10864 | 60254 |
| Redundancy | 6.0 | 7.18 |
| Completeness (%) | 97.4 | 99.92 |
| R_merge_^#^ | 7.2 | 8.8 |
| Average I/σ(I) | 13.04 | 12.23 |
| Wilson B (Å^2^) | 73.7 | 35.39 |
| Mosaicity | 0.140 | 0.128 |
| **Refinement statistics** | | |
| No. of reflections in working set | 6335 | 59204 |
| R value (%)^##^ | 25.22 | 21.66 |
| R_free_ value (%)^###^ | 30.97 | 26.00 |
| RMSD bond length (Å) | 0.008 | 0.015 |
| RMSD angle (°) | 0.001 | 0.001 |
| No. of atoms in AU | 1058 | 4660 |
| No. of protein atoms in AU | 1007 | 4274 |
| No. of water molecules in AU | 49 | 356 |
| No. of ions in AU | 0 | 10 |
| No. of zinc ions | 0 | 4 |
| No. of phosphate ions | 0 | 6 |
| Mean B value (Å^2^) | 27.55 | 21.69 |
| Ramachandran plot statistics: |  |  |
| - Residues in favoured regions (%) | 100/100 | 100/100 |
| - Residues in allowed regions (%) | 84/100 | 94.44/100 |
| PDB code | 8Q2G | XXXX |

The data in parentheses refer to the highest-resolution shell.

^#^ R_merge_ = (|I_hkl_ -
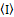

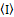
|)/I_hkl_, where the average intensity
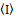

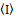
is taken over all symmetry equivalent measurements and I_hkl_ is the measured intensity for any given reflection

^##^ R-value = ||F_o_| - |F_c_||/|F_o_|, where F_o_ and F_c_ are the observed and calculated structure factors, respectively.

^###^ R_free_ is equivalent to R value but is calculated for 5 % of the reflections chosen at random and omitted from the refinement process.
