# Supplementary material for: Development of novel antimicrobials with engineered endolysin LysECD7-SMAP to combat Gram-negative bacterial infections
Source: J Biomed Sci. 2024 Jul 24;31:75. doi: 10.1186/s12929-024-01065-y (PMC11267749; doi:10.1186/s12929-024-01065-y)
Supplement: Supplementary file 2 — Additional File 2. LysECD7 structural traits and characteristics. [file 12929_2024_1065_MOESM2_ESM.docx]

**LysECD7 structure contains sequences with putative antimicrobial traits.**

Native LysECD7 endolysin sequence belongs to peptidase M15 superfamily (cl38918, cd14845 subfamily) as defined by the Conserved Domains Database (https://www.ncbi.nlm.nih.gov). This superfamily summarizes zinc-binding metallopeptidases which are mostly carboxypeptidases and dipeptidases. The cd14845 subfamily relates to L-Ala-D-Glu endopeptidases, typified by peptidase from bacteriophage T5, Ply500 peptidases from *Listeria* phage A500, LysB4 from *Bacillus cereus*-targeting phage B4, cleaving the peptide bonds between L-Ala and D-Glu residues of bacterial cell wall peptidoglycan, leading to cell lysis. The LysECD7 core cleft includes three α-helices (α2 residues 43-50, α3 res. 61-64, and α5 res. 112-116) and three β-strands forming one β-sheet (res. 34-38, 67-72, and 117-119). Catalytic residues His62, Asp69 and His117 are located in α3, β2 and β3 correspondingly, while Asp114 in α5. The only catalytically active residue, located in unstructured loop region connecting β1 and α2 is Arg41, proposing the flexibility and plasticity for catalysis. Also, loop connecting α4 and α5 (res. 100-107) is saturated with glycines, which makes it possible to classify it as a mobile one.

Despite the impeded access of endolysins to peptidoglycan layer in Gram-negative bacteria, protected by a robust outer membrane, their cleavage activity from outside followed by the death of bacterial cells is well documented. Obviously, this mode of action is associated not only with the catalytic properties of the enzyme, but requires additional functional domains. At the moment, the leading role in this process is attributed mainly to the presence of specific structures within molecules such as β-hairpins and AMP-like elements with high local content of positively charged residues^1,2^*.* These regions present significantly higher net charge per residue (NCPR), hydrophobicity, and average hydrophobic moment (HM)^2^. On the other hand, a combination of low overall hydrophobicity and large net charge are characteristic structural features of “natively unfolded” or intrinsically disordered regions (IDR) – polypeptides that are not likely to form a defined 3D-structure, but actively participate in diverse protein mediated functions^3,4^. High occurrence of IDR in viral proteins is established and associated with increased structural flexibility and effective ways to interact with the components of the host cells^5^. IDRs are inclined to binding-induced folding during the interaction to their partners or under specific conditions adopting diverse assemblies which allow to form complexes with some structural heterogeneity^6^. In the case of gram-negative-targeting endolysins, this may extend the specificity of enzymes, allowing the activity against several bacterial species with moderate modifications in cell wall structure.

For LysECD7 three IDR regions were predicted (Fig. S1a,c), however, two of them (IDR-1 and IDR-3), are located within its terminal regions which is typical^7^ and correspond to terminal loops. IDR-2 is located in the core of sequence and includes the entire α2-helix and unstructured loop enriched with positively charged residues (lysins and arginine) along the inner surface, one of which (K56) together with K113 are located at both ends of the active site cleft at 7.5 Å distance (Fig. S1d,e).

**
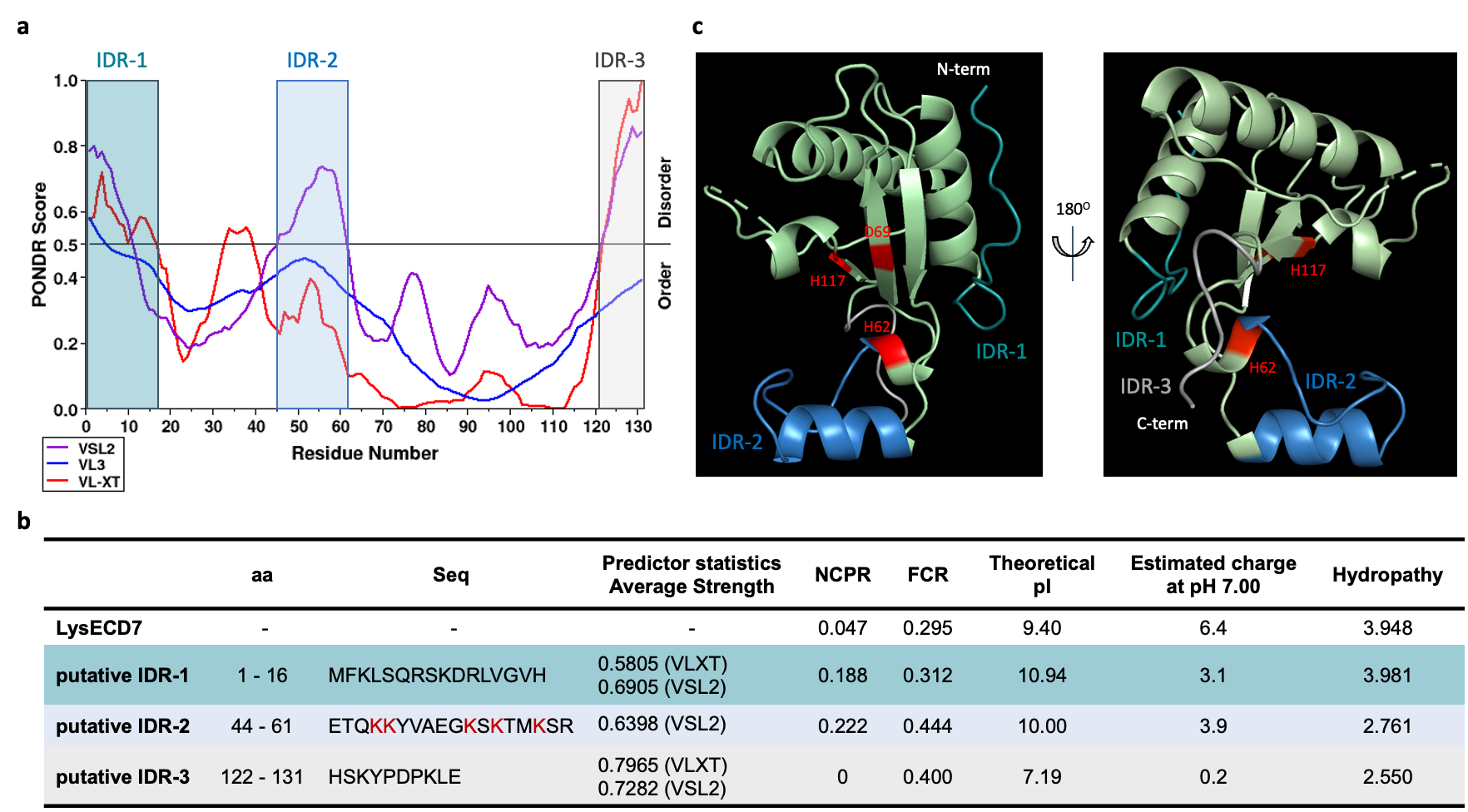
**

**
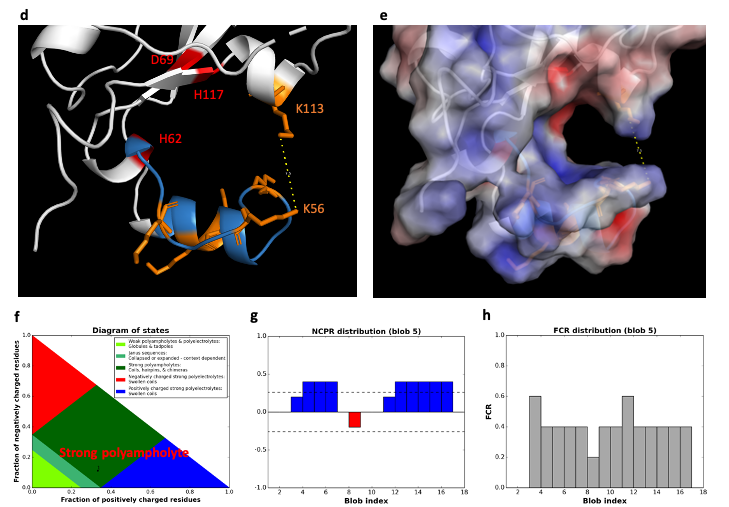
**

**Figure S1. Prediction of intrinsically-disorder regions (IDR) in LysECD7.** **a** IDR prediction with three different algorithms. **b** IDRs’ characteristics as predicted by PONDR software. **c** Localization of the potential intrinsically-disorder regions in LysECD7 structure. **d** Structure of IDR-2 and its counteraction with enzyme active center. Orange indicates the arrangement of lysine residues. **e,** Electrostatic potentials of IDR-2 surface. Negatively charged surface is colored in red, positively charged surface - in blue. **f-h** Sequence parameters associated with putative IDR-2 sequence: Das-Pappu phase plot (f), net charge per residue distribution (NCPR, g), and fraction of charged residues (FCR, h).

For 18aa-long IDR-2 predicted features (Fig. S1b) include the presence of multiple charged groups that define the relatively high net charge (0.222) and extreme isoelectric point value (pI = 10.0), combined with a low content of hydrophobic amino acid residues (hydropathy = 2.761), serving as a prerequisite for the absence of compact structure in proteins under physiological conditions. As defined by Das-Pappu diagram of states, IDR-2 is a strong polyampholyte, associated with typical conformations of coils, hairpins, chimeras^8^. Previously, homologous regions were predicted, for example, for EndoT5 bacteriophage peptidas^9^, proposing this feature to be common for at least L-Ala-D-Glu endopeptidases. Additionally, *in silico* sequence activities’ prediction using AxPEP Server and AMPfun web server identified antibacterial peptide targeting Gram-positive and Gram-negative bacteria located in IDR-2 (AmPEP and Deep-AmPEP30 instruments, scores 0.730000 for QKKYVAEGKSKTMK peptide and 0.578211 for QKKYVAEGK correspondingly) and (AMPfun web server Scores 0.578211 QKKYVAEGK and 0.8973 for QKKYVAEGKSKTMK). Alternatively, no AMPs were predicted be AMP Scanner Ver.2. Thus, we propose sequence of IDR-2 to be the most likely candidate as additional functional domain in LysECD7 and hybrid enzyme LysECD7-SMAP, determining their broad spectra of action.

**
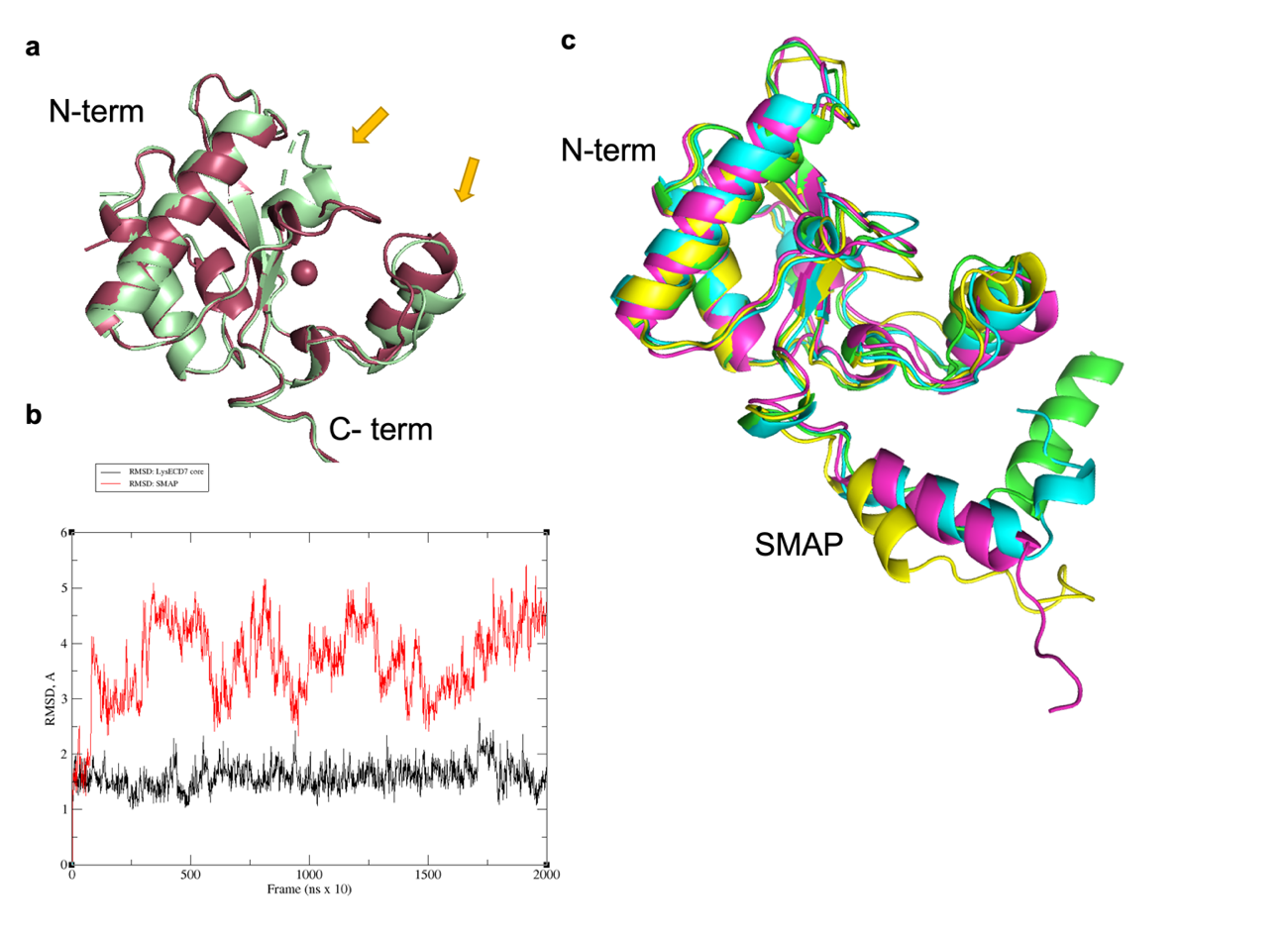
**

**Figure S2. Comparison of LysECD7 and LysECD7-SMAP structures. a** Superimposed structures of LysECD7 (pale green) and LysECD7-SMAP (burgundy). Arrows indicate structural shifts in peptidoglycan-binding pocket in native and hybrid molecules. **b** 200 ns simulation of LysECD7-SMAP model. SMAP part (red) demonstrates rapidly changing structure while «core» protein domain without SMAP (black) appears to remain stable. **c** LysECD7-SMAP simulation frames according to initial model (green), 50 ns (cyan), 100 ns (magenta) and 200 ns (yellow).

**References**

1. Premetis, G. E., Stathi, A., Papageorgiou, A. C. & Labrou, N. E. Characterization of a glycoside hydrolase endolysin from Acinetobacter baumannii phage AbTZA1 with high antibacterial potency and novel structural features. *FEBS J* **290**, (2023).

2. Vázquez, R., Blanco-Gañán, S., Ruiz, S. & García, P. Mining of Gram-Negative Surface-Active Enzybiotic Candidates by Sequence-Based Calculation of Physicochemical Properties. *Front Microbiol* **12**, 660403 (2021).

3. Van Der Lee, R. *et al.* Classification of intrinsically disordered regions and proteins. *Chem Rev* **114**, 6589–6631 (2014).

4. Uversky, V. N., Gillespie, J. R. & Fink, A. L. Why are ‘natively unfolded’ proteins unstructured under physiologic conditions? *Proteins: Structure, Function, and Genetics* **41**, 415–427 (2000).

5. Tokuriki, N., Oldfield, C. J., Uversky, V. N., Berezovsky, I. N. & Tawfik, D. S. Do viral proteins possess unique biophysical features? *Trends Biochem Sci* **34**, 53–59 (2009).

6. Uversky, V. N. Intrinsically disordered proteins and their ‘Mysterious’ (meta)physics. *Front Phys* **7**, 10 (2019).

7. Uversky, V. N. The most important thing is the tail: multitudinous functionalities of intrinsically disordered protein termini. *FEBS Lett* **587**, 1891–1901 (2013).

8. Das, R. K. & Pappu, R. V. Conformations of intrinsically disordered proteins are influenced by linear sequence distributions of oppositely charged residues. *Proc Natl Acad Sci U S A* **110**, 13392–13397 (2013).

9. Prokhorov, D. A., Mikoulinskaia, G. V., Molochkov, N. V., Uversky, V. N. & Kutyshenko, V. P. High-resolution NMR structure of a Zn2+-containing form of the bacteriophage T5 L-alanyl-D-glutamate peptidase. *RSC Adv* **5**, 41041–41049 (2015).
