# Supplementary material for: Development of novel antimicrobials with engineered endolysin LysECD7-SMAP to combat Gram-negative bacterial infections
Source: J Biomed Sci. 2024 Jul 24;31:75. doi: 10.1186/s12929-024-01065-y (PMC11267749; doi:10.1186/s12929-024-01065-y)
Supplement: Supplementary file 3 — Additional File 3. Supplementary figure S3. LysECD7-SMAP interactions with components of bacteria. [file 12929_2024_1065_MOESM3_ESM.docx]

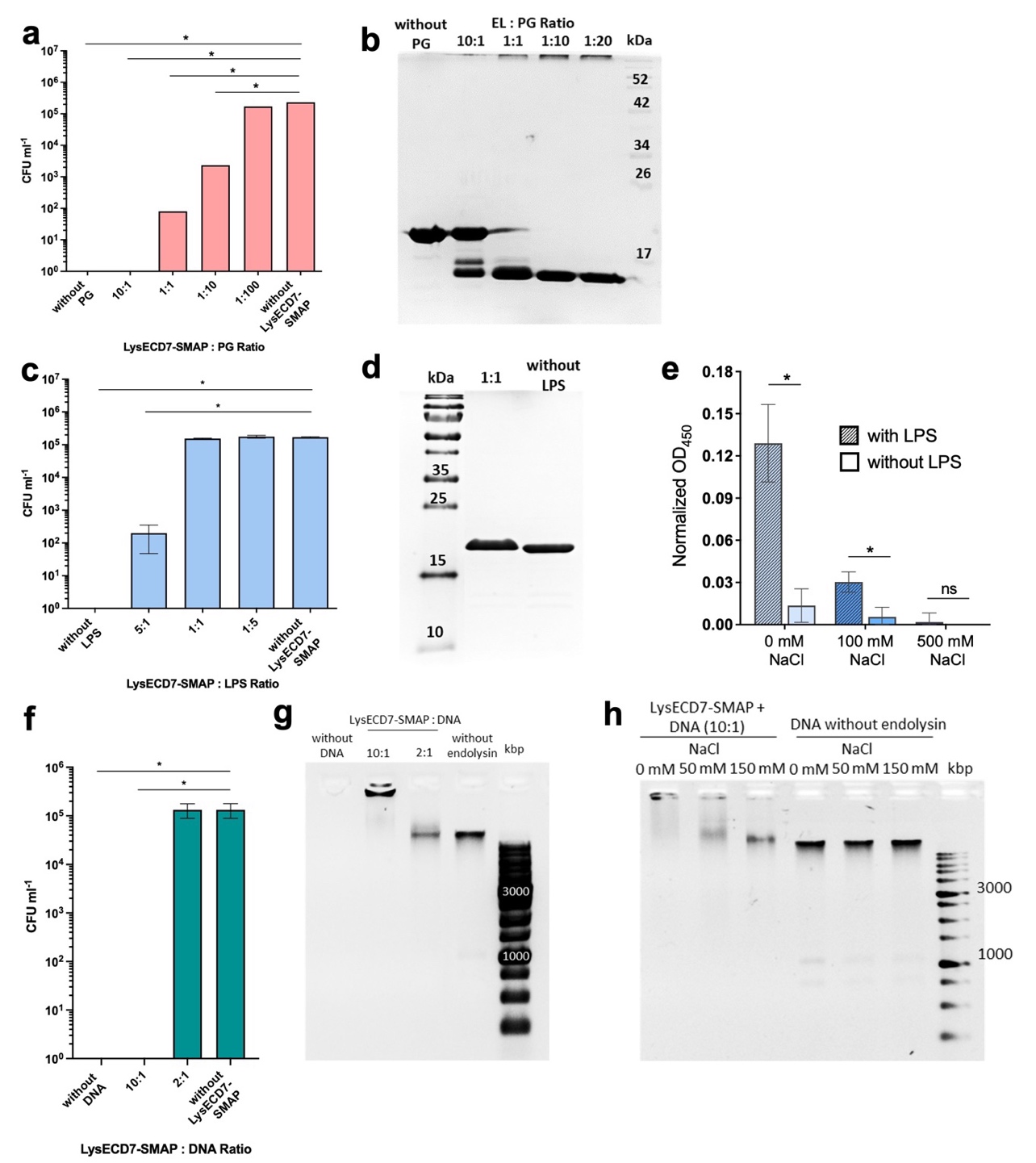


**Figure S3. LysECD7-SMAP interactions with components of bacteria**. **a** Antibacterial activity of LysECD7-SMAP (10 µg ml^-1^) in the presence of bacterial peptidoglycan (PG). Data are shown as mean ± SD (one-way ANOVA). **b** SDS-PAGE of LysECD7-SMAP pre-incubated for 30 min with PG. **c** Antibacterial activity of LysECD7-SMAP (5 µg ml^-1^) in the presence of bacterial lipopolysaccharides (LPS). Data are shown as mean ± SD (one-way ANOVA). **d** SDS-PAGE of LysECD7-SMAP pre-incubated for 30 min with LPS. **e,** ELISA with LPS. **f** Antibacterial activity of LysECD7-SMAP (1 µg ml^-1^) in the presence of bacterial DNA. Data are shown as mean ± SD (one-way ANOVA). **g** Agarose electrophoresis of LysECD7-SMAP pre-incubated for 30 min with bacterial DNA. **h** Agarose electrophoresis of LysECD7-SMAP pre-incubated for 30 min with bacterial DNA in the presence of different concentrations of NaCl.
