# Supplementary material for: Development of novel antimicrobials with engineered endolysin LysECD7-SMAP to combat Gram-negative bacterial infections
Source: J Biomed Sci. 2024 Jul 24;31:75. doi: 10.1186/s12929-024-01065-y (PMC11267749; doi:10.1186/s12929-024-01065-y)
Supplement: Supplementary file 4 — Additional File 4. Additional data of LysECD7-SMAP efficacy studies. [file 12929_2024_1065_MOESM4_ESM.docx]

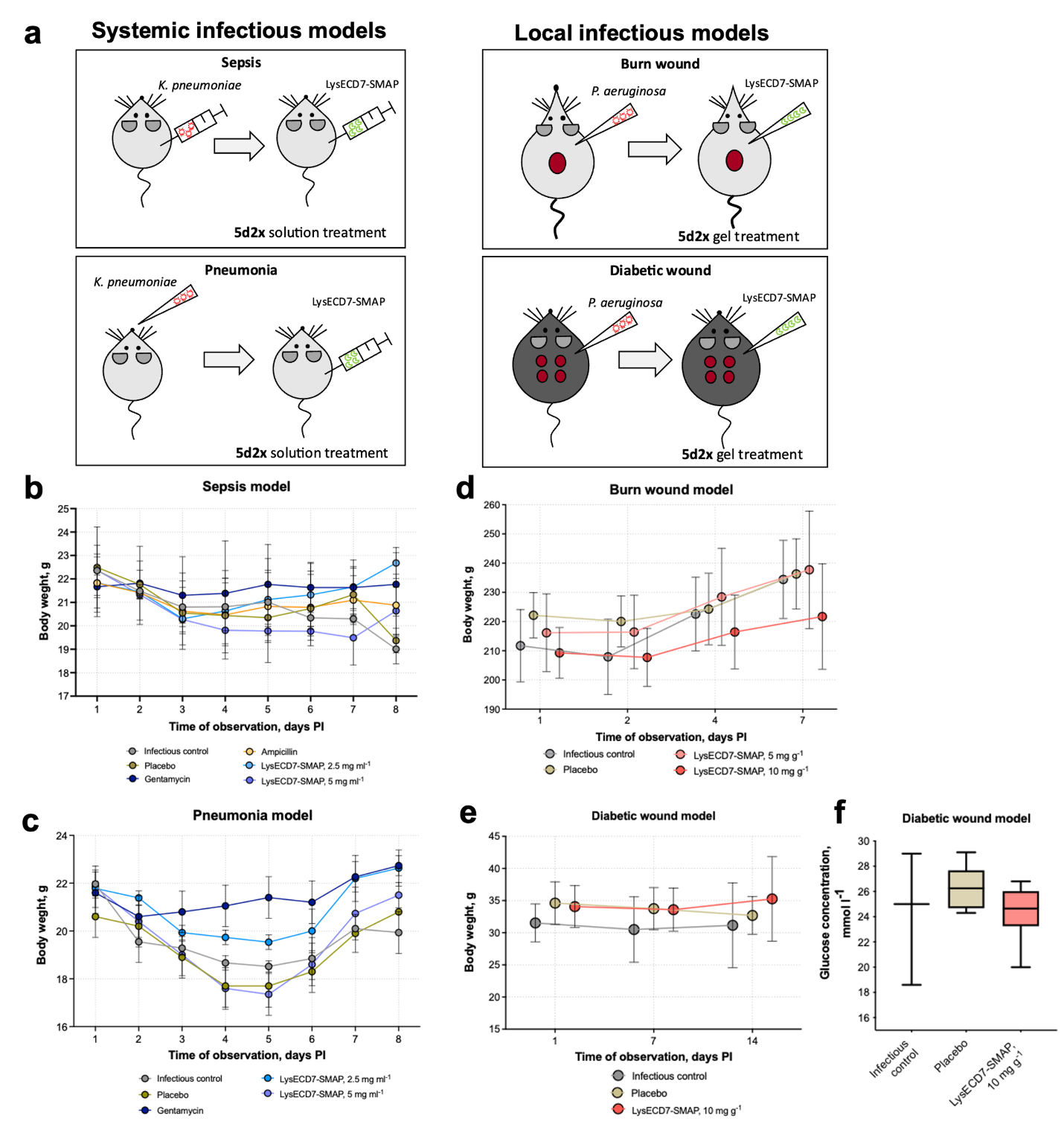


**Figure S4. *In vivo* efficacy studies of LysECD7-SMAP dosage forms. a** Animal models schematic workflow. **b** Mice weight changes in *K. pneumoniae* caused sepsis model. **c** Mice weight changes in *K. pneumoniae* caused pneumonia model. **d** Rats weight changes in *Pseudomonas aeruginosa* caused burn wound infection model. **e** Mice weight changes in *P. aeruginosa* caused diabetes-associated wound infection model. Data are shown as mean ± SD. **f** Blood glucose (non-fasted) concentration in mice before the beginning of experimental procedures.

**LysECD7-SMAP based gel modifies microbiological** **composition of chronic wounds in diabetes-associated infection model**. Prior to injury and infection all animals were tested for diabetes and the median glucose concentrations were 21.8–26.2 mmol l^-1^ depending on the group (Fig. S4f). Also, no significant differences in body mass between the groups were detected (Fig. S4e), although there was a tendency for less weight accumulation in the IC group compared to the placebo and LysECD7-SMAP treatment. Wounds surface examination revealed an insufficient regeneration phase and decreased regenerative reaction, indicating the presence of trophic disorders associated with diabetes.

Although the animals used in the study were SPF for primary pathogens and opportunists, they were not housed under SPF conditions during the experiment, which means availability towards external environmental microbiome as well as normal microbial flora colonization due to animals diabetes and obesity-induced dislocation of normal bacterial sites. To define the bacterial spectra cultivated in samples, the characteristic colonies from wound swabs, dermal graft and blood were genotyped using 16S rRNA sequencing (Table S3). In the IC group, the vast majority of CFUs in all samples (wound swabs, dermal graft homogenates and blood) was *P. aeruginosa*, corresponding to the sequence of the infectious agent, which was also confirmed by the characteristic fluorescence of colonies under UV light, due to the production of pyoverdine and a blue green pigmentation (pyocyanin production). No target *P. aeruginosa* was detected in the swabs and blood of the placebo and LysECD7-SMAP groups, however, it was found in dermal graft homogenates, which indicates the penetration of bacteria into the deeper layers of the wound defects.

**Table S3.** Bacterial species identified in wound swabs, dermal graft and blood samples at day 14^th^ PI in diabetic wound infection model. G + - Gram-positive species, G- - Gram-negative species.

|  | **Group** | **Microorganism** | **Gram** **staining** | **Per. Ident** |
| --- | --- | --- | --- | --- |
| Wound swabs | | | | |
| 1 | Infectious control | ***Pseudomonas aeruginosa*** | G - | 100.0% |
| 2 | Placebo | *Enterobacter cloacae complex* | G - | 99.68% |
|  |  | *Rahnella sp./Ewingella sp.* | G - | 100.0% |
|  |  | *Staphylococcus saprophyticus (xylosus)* | G + | 100.0% |
|  |  | *Staphylococcus sciuri* | G + | 100.0% |
| 3 | LysECD7-SMAP, 10 mg g^-1^ | *Pseudomonas fluorescens complex* | G - | 100.0% |
|  |  | *Staphylococcus sciuri* | G + | 100.0% |
| Dermal graft | | | | |
| 1 | Infectious control | ***Pseudomonas aeruginosa*** | G - | 100.0% |
| 2 | Placebo | ***Pseudomonas aeruginosa*** | G - | 100.0% |
|  |  | *Escherichia coli* | G - | 95.7% |
|  |  | *Rahnella sp./Ewingella sp.* | G - | 100.0% |
|  |  | *Staphylococcus sciuri* | G + | 99.86 |
| 3 | LysECD7-SMAP, 10 mg g^-1^ | ***Pseudomonas aeruginosa*** | G - | 100.0% |
|  |  | *Staphylococcus sciuri* | G + | 99.93% |
|  |  | *Staphylococcus xylosus* | G + | 100% |
| Blood | | | | |
| 1 | Infectious control | ***Pseudomonas aeruginosa*** | G - | 100.0% |
| 2 | Placebo | *Escherichia coli* | G - | 99.85% |
|  |  | *Rahnella sp./Ewingella sp.* | G - | 100.0% |
|  |  | *Staphylococcus xylosus* | G + | 100% |
| 3 | LysECD7-SMAP, 10 mg g^-1^ | *Escherichia coli* | G - | 99.85% |
|  |  | *Staphylococcus sciuri* | G + | 99.93% |

The vehicle-treated group showed greater diversity of cultivated bacteria (*Enterobacter cloacae* complex, *E. coli*, *Rahnella* sp./*Ewingella americana*, *S. saprophyticus*, *S. sciuri*) comparing to LysECD7-SMAP (*S. saprophyticus*, *S. sciuri*, *E. coli* and *P. fluorescens* сomplex). Most species were observed in the swabs of the placebo group, where Gram-negative Enterobacteriaceae (*Enterobacter cloacae* complex, *Rahnella* sp./*Ewingella americana*), as well as Gram-positive coagulase-negative Staphylococcal species (*S. saprophyticus, S. sciuri*) were identified. The LysECD7-SMAP swabs contained CFUs of *S. sciuri* and *P. fluorescens* сomplex, but not *P.* *aeruginosa.* Similar results were obtained for dermal graft homogenates: *Staphylococcus* species in placebo and LysECD7-SMAP groups and multiple Enterobacteriaceae colonies (*Escherichia coli, Rahnella* sp./*Ewingella* sp.) in placebo. In addition, grafts of all groups contained target *P. aeruginosa*, which indicates the penetration of bacteria into the deeper layers of the wound defects. Analysis of murine blood samples after the gel treatment revealed *E. coli* и *S. sciuri*, and after the vehicle treatment – *E. coli* и *S. sciuri* and *Rahnella* sp./*Ewingella* sp. No target infectious agent *P. aeruginosa* was detected in blood of placebo or LysECD7-SMAP treated animals.

The bacterial composition, in most cases, is represented with commensals inhabiting the skin of animals, and the coagulase-negative Staphylococci are the most commonly isolated skin species in mice housed under SPF conditions^1^. It is worth noting that during the treatment with placebo or endolysin-based gel, *P.* *aeruginosa* did not spread to the bloodstream, which means that the risk of sepsis and the development of a systemic infection is reduced. Thus, it can be seen from the sequencing results that by the 14^th^ day, the wound flora in LysECD7-SMAP and vehicle treated groups is formed mainly by Gram-positive and commensal species, but not by the target infectious agent, which is identified in dermal graft samples only, which indicates the effectiveness of endolysin-based gel administration for wound healing process.

**LysECD7-SMAP topical antimicrobial is active in a burn wound infection model of Wistar rats.** Regeneration, epithelialization and wound healing begun with the 3^rd^ day PI in LysECD7-SMAP, 10 mg g^-1^, with the wound closure up to 55 mm^2^ (25.9% of initial wound) by the 7^th^ day (Fig. S5a). For other groups less pronounced effects were observed with average closure of burn wound area by 12.5, 22.0 and 13.2 mm^2^ (5.6, 9.8 and 5.6%) in untreated, vehicle-treated and 5 mg g^-1^ gel groups correspondingly. Thus, no healing effect was estimated for the 5 mg g^-1^ gel preparation and placebo comparing to control group while LysECD7-SMAP in concentration of 10 mg g^-1^ significantly reduced the wound area.


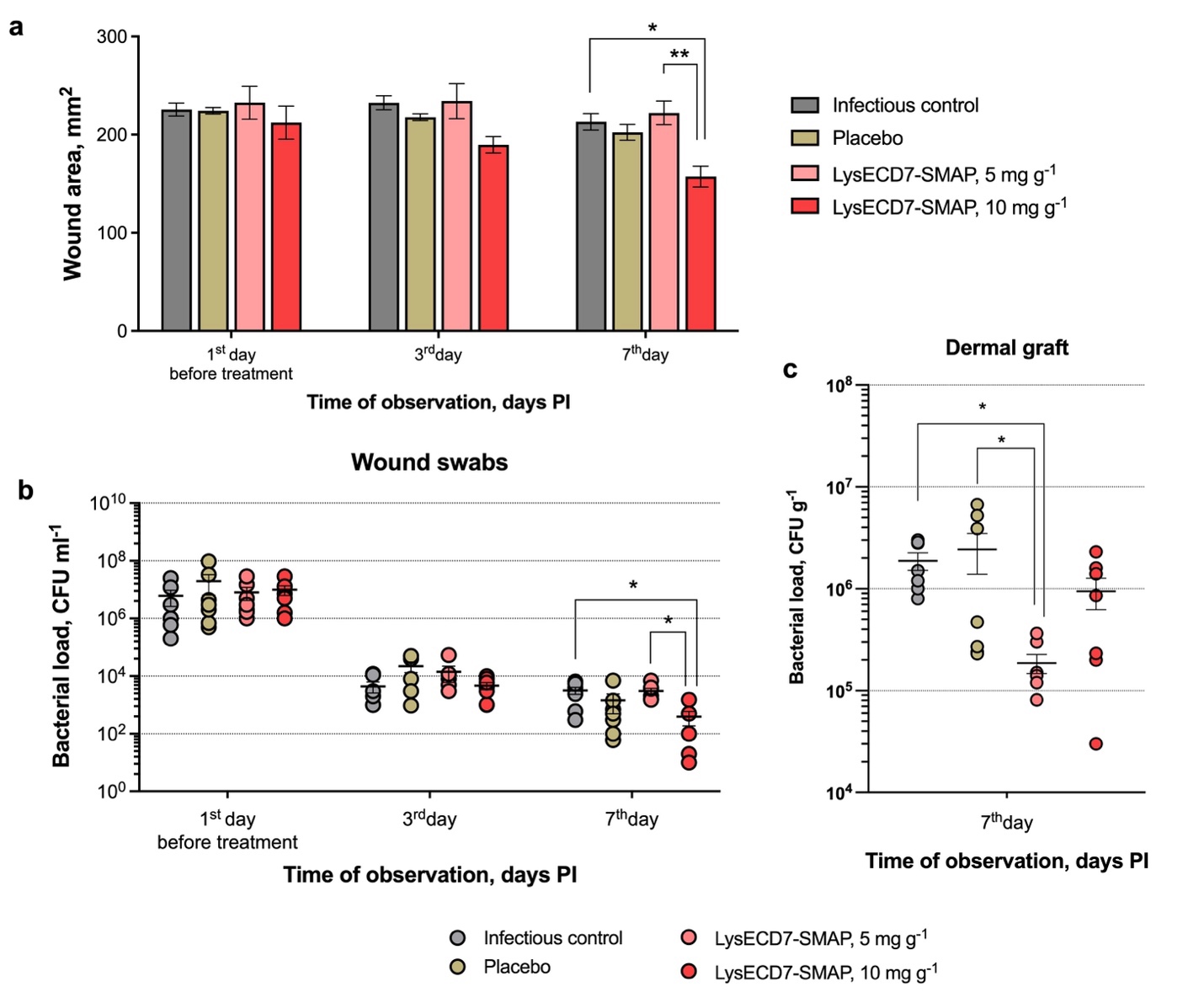


**Figure S5. Burn wound infection model of Wistar rats infected with *P. aeruginosa* 38-16. a** Wound-closure course in the investigated groups. **b, с** Bacterial loads of wound swabs (b) and dermal graft homogenates (c) during and after the treatment. Data are shown as the mean ± SEM. Significant differences are shown as asterisks, otherwise no statistical difference is found (Kruskal-Wallis test, Dunn’s multiple comparisons test), * - p < 0.05, ** - p < 0.005, *** - p < 0.001.

Unlike the IC group in chronic wound infection model, absence of bacteria in animals’ spleens was observed, indicating no transition of infection from local to generalized form. During the observation period, median load in wound swabs reduced from 2×10^5^ – 3×10^7^ CFU ml^-1^ before the treatment to 2.0×10 – 7.0×10^3^ CFU ml^-1^ on the 7^th^ day in all groups studied, including the untreated control, indicating pronounced self-recovery of animals after the infection with *P. aeruginosa* (Fig. S5b, c). The epicutaneous application of endolysin-based gel in concentration of 10 mg g^-1^ reduced median bacterial load by more than 4 orders of magnitude, of initial load while in control group the reduction was about 3 orders. On the contrary, decolonization of dermal grafts was more pronounced in 5 mg g^-1^ gel.

**References**

1. Tavakkol, Z. *et al.* Resident Bacterial Flora in the Skin of C57BL/6 Mice Housed under SPF Conditions. *J Am Assoc Lab Anim Sci* **49**, 588 (2010).
