# Supplementary material for: Development of novel antimicrobials with engineered endolysin LysECD7-SMAP to combat Gram-negative bacterial infections
Source: J Biomed Sci. 2024 Jul 24;31:75. doi: 10.1186/s12929-024-01065-y (PMC11267749; doi:10.1186/s12929-024-01065-y)
Supplement: Supplementary file 5 — Additional File 5. LysECD7-SMAP in vitro toxicity tests. [file 12929_2024_1065_MOESM5_ESM.docx]

**LysECD7-SMAP do not reveals cytotoxic effects or resistance development.** High activity against prokaryotic cells, as well as the presence of a cationic charged C-terminal peptide, suspect that the enzyme may be active against eukaryotic cells as well, limiting its use in clinical settings. However, *in vitro* cytotoxicity assays against human red blood cells and eukaryotic cells showed that LysECD7-SMAP administration in concentration up to 1 mg ml^-1^ has no toxic effect (Fig. S6a). The hemolysis percent was low and did not exceed 5%, that is unsignificant. Viability of HEK293 cells varied from 93.7% in 16 µg ml^-1^ concentration to 139.3% in 1 mg ml^-1^ concentration, and minimal cells disaggregation was shown in 1 mg ml^-1^ concentration (Fig. S6b, c).

**
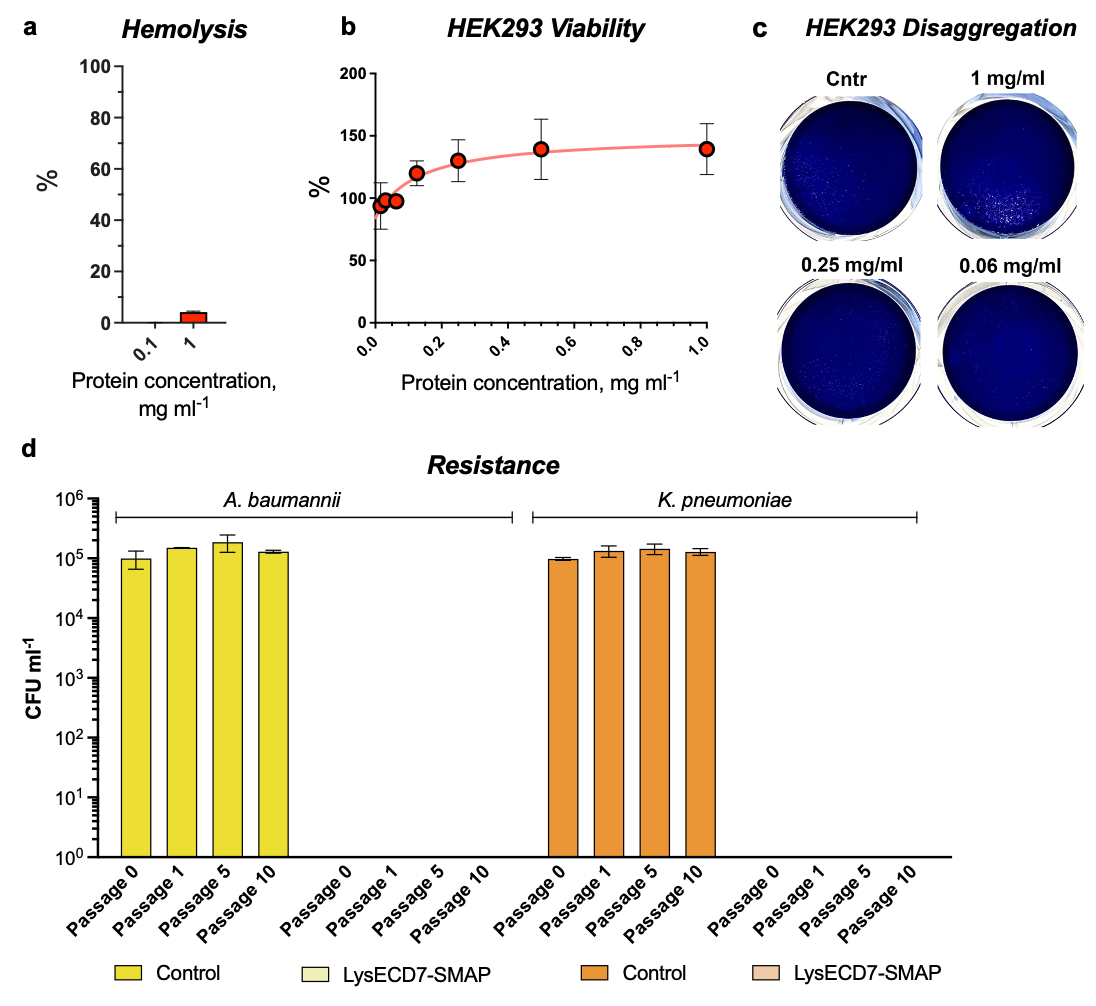
**

**Figure S6. LysECD7-SMAP *in vitro* safety characteristics.** **a** Hemolytic activity of LysECD7-SMAP against human red blood cells after 1 h exposure. **b** LysECD7-SMAP cytotoxic activity on the viability of HEK293 cells was determined with an MTT assay after 1 h exposure. The mean values with SD are shown, all experiments were performed in triplicate. **c** HEK293 cell-disagregation assay after 48 h of incubation with different concentrations of LysECD7-SMAP. **d** Bactericidal activity of LysECD7-SMAP against initial and 10 serials passed strains of *A. baumannii* and *K. pneumoniae* clinical isolates.

The resistance development measured with the serial passage experiments was studied on *A. baumannii* and *K. pneumoniae* clinical isolates in a plate lysis assay. After 10 passages we did not detect difference in antibacterial activity of LysECD7-SMAP against initial and passed strains, proposing that no resistance is developed towards endolysin (Fig. S6d).
